# Population-specific physiological response profiles characterize salinity tolerance in the Antarctic extremophile *Colobanthus quitensis*

**DOI:** 10.3389/fpls.2026.1909187

**Published:** 2026-08-10

**Authors:** Marely Cuba-Díaz, Eduardo Fuentes-Lillo, Yadiana Ontivero, Darío Navarrete-Campos, Francisca Arroyo, Thiare Aguilera, Gerardo Alejo-Jacuinde, Luis Herrera-Estrella

**Affiliations:** 1 Laboratorio de Biotecnología y Estudios Ambientales, Departamento de Ciencias y Tecnología Vegetal, Escuela de Ciencias y Tecnologías, Universidad de Concepción Campus Los Ángeles, Los Ángeles, Chile; 2 Programa de Ciencia Antártica y Subantártica (PCAS), Universidad de Concepción, Concepción, Chile; 3 Laboratorio de Invasiones Biológicas (LIB), Facultad de Ciencias Forestales, Universidad de Concepción, Concepción, Chile; 4 Instituto de Ecología y Biodiversidad (IEB), Concepción, Chile; 5 Facultad de Ingeniería, Instituto de Ciencias Aplicadas, Centro de Investigación e Innovación, Universidad Autónoma de Chile, Huechuraba, Chile; 6 Institute of Genomics for Crop Abiotic Stress Tolerance, Department of Plant and Soil Science, Texas Tech University, Lubbock, TX, United States; 7 Unidad de Genómica Avanzada/Langebio, Centro de Investigación y de Estudios Avanzados del Instituto Politécnico Nacional, Irapuato, Guanajuato, Mexico

**Keywords:** Antarctic vascular plant, *Colobanthus quitensis*, compatible-solute responses, oxidative stress, physiological integration, population-specific responses, salinity tolerance, tissue water status

## Abstract

**Introduction:**

Extremophile plant species provide valuable systems for understanding how salinity tolerance varies among populations originating from contrasting environments. The Antarctic vascular plant Colobanthus quitensis, is widely distributed from temperate South America to the maritime Antarctic and represents an useful model for investigating population-level physiological variation under environmental constraints. However, the physiological basis of variation in salinity tolerance among population remains poorly understood.

**Methods:**

Here, we evaluated morphophysiological and biochemical responses of five geographically and environmentally contrasting C. quitensis populations exposed for 60 days to a NaCl concentrations (0–200 mM) under controlled in vitro conditions. Dry biomass, tissue water content, root-to-shoot length ratio, proline, total soluble sugars, lipid peroxidation, and antioxidant enzyme activities were examined using univariate analyses, standardized response ratios, principal component analysis, an exploratory integrated response score, and observed-variable path analysis.

**Results:**

Population, NaCl concentration, and their interaction significantly affected most evaluated traits, demonstrating strongly population-dependent responses. Proline accumulation increased with salinity in most populations, whereas total soluble sugars and antioxidant enzyme activities showed contrasting trajectories. Tissue water content was generally maintained or slightly increased, indicating sustained tissue hydration during prolonged NaCl exposure. Multivariate and integrated analyses showed that populations differed in the magnitude and combination of growth-related, water-status, compatible-solute-related, antioxidant, and oxidative-damage responses. Under high salinity, averaged across 150 and 200 mM NaCl, the inland population pV showed the highest exploratory integrated response score, followed by pH, whereas pPA showed the lowest score. The observed-variable path analysis identified conditional associations among NaCl concentration, proline, antioxidant enzyme activity, MDA, root-to-shoot length ratio, and dry biomass, but did not support a simple linear relationship between antioxidant activation, oxidative damage, and biomass maintenance.

**Discussion:**

Overall, salinity tolerance in C. quitensis was associated with population-specific physiological configurations rather than a single common response profile. The absence of a simple correspondence between these profiles and broad present-day habitat categories further suggests that ecological and evolutionary histories may contribute to physiological diversification within the species.

## 1 Introduction

Soil salinity is one of the most pervasive environmental constraints limiting plant growth and productivity worldwide. Salinity imposes a complex stress scenario involving osmotic imbalance, ion toxicity, oxidative stress, and metabolic disruption, ultimately affecting growth, water relations, and cellular homeostasis. Plant responses to salinity therefore rely on the coordinated regulation of multiple physiological and biochemical processes operating simultaneously under stress conditions, rather than on the activation of a single protective mechanism (Busoms et al., 2023; Waheed et al., 2024). Increasing evidence indicates that salinity tolerance emerges from the integration and coordination of traits associated with osmotic adjustment, ion homeostasis, oxidative stress regulation, and growth maintenance, whose relative contributions may vary among genotypes, populations, and environmental contexts (Augstein and Carlsbecker, 2022). In this study, salinity tolerance is defined operationally as the capacity to maintain growth and measured physiological status while limiting visible and oxidative damage during sustained NaCl exposure relative to non-saline control conditions. Because post-stress recovery was not evaluated, resilience is not used to characterize the responses measured in this experiment.

Plant responses to salinity commonly involve osmotic adjustment through compatible solute accumulation, modulation of antioxidant systems, maintenance of tissue water balance, regulation of ion homeostasis, and changes in root and shoot growth. Many of these processes are also shared with other osmotic stresses, including drought and dehydration, resulting in substantial physiological overlap and the potential for cross-tolerance among stress responses (Angon et al., 2022; Malambane et al., 2023; Cao et al., 2024). However, the relative contribution of these mechanisms is not necessarily identical across individuals or populations of the same species (Shelake et al., 2022). Growing evidence indicates that intraspecific variation among populations originating from contrasting environments may be expressed through distinct physiological strategies, in which different combinations of traits and stress-response mechanisms converge on similar levels of salinity tolerance (Augstein and Carlsbecker, 2022; Schrieber et al., 2023). Consequently, populations exposed to similar environmental constraints may not necessarily rely on identical physiological responses, highlighting the need for integrative approaches that evaluate how multiple traits are coordinated under stress conditions (Zhu et al., 2025).

The Antarctic vascular plant *Colobanthus quitensis* (Kunth) Bartl. provides a valuable natural system for investigating population-level physiological variation across contrasting and environmentally challenging habitats. One of only two native vascular plant species in Antarctica, *C. quitensis* has a broad latitudinal distribution extending from temperate South America to the maritime Antarctic, where it persists under multiple environmental constraints, including freezing temperatures, intense radiation, dehydration, and saline influence associated with coastal environments and marine aerosols (Cavieres et al., 2016; Biersma et al., 2020). Previous studies have documented substantial phenotypic plasticity, metabolic flexibility, and population-level variation in responses to abiotic stressors across its distribution range (Gianoli et al., 2004; Cuba-Díaz et al., 2017b; Bertini et al., 2022; Min et al., 2024).

The five populations examined here represent qualitatively contrasting habitat contexts. The Antarctic populations from Arctowski and Punta Hannah occur in coastal ice-free environments exposed to marine influence, although their local settings differ in topography and biotic disturbance. Punta Hannah is characterized by abundant seabird and marine-mammal activity, which may contribute to localized physical disturbance and nutrient enrichment. Among the Magellanic populations, La Marisma occurs in a low-elevation coastal salt marsh subject to periodic seawater inundation, whereas Laredo represents a disturbed coastal site exposed to marine influence, although tidal flooding was not quantified at this site. In contrast, La Vega is located in an inland grassland without direct marine influence, where seasonal water limitation and grazing activity represent potential environmental pressures. These ecological descriptors are based on published regional information, collection records, and field observations rather than on standardized environmental monitoring at each collection site. Accordingly, they provide context for population selection but are not treated as directly measured explanatory variables. Accordingly, the physiological differences observed here are interpreted as population-level variation, while local adaptation and environmental-history effects remain biologically relevant hypotheses rather than direct conclusions of the present experiment.

Recent experimental evidence has shown that populations of *C. quitensis* originating from contrasting habitats differ markedly in their responses to salinity, including relatively high salinity tolerance in inland populations lacking direct present-day exposure to marine-derived salinity (Cuba-Díaz et al., 2025). This pattern indicates that contemporary exposure to saline environments alone does not reliably predict salinity tolerance under experimental NaCl exposure. More generally, responses to salinity and other forms of osmotic stress may involve partially shared physiological mechanisms, including osmotic adjustment, maintenance of tissue water status, and regulation of oxidative damage (Cao et al., 2024; Shelake et al., 2022; Sulaiman et al., 2026). Despite increasing evidence of population-level variation in stress responses, the physiological basis of these contrasting response patterns remains poorly understood. In particular, it remains unclear whether populations exhibiting comparable or partially overlapping salinity tolerance rely on similar physiological responses or on alternative patterns of physiological coordination involving compatible-solute-related responses, antioxidant activity, oxidative damage, relative root and shoot growth, maintenance of tissue water status, and ion-related responses. Addressing this question may provide important insights into how different levels and profiles of salinity tolerance are associated with distinct combinations of physiological responses among populations inhabiting contrasting environments.

In this study, we evaluated the morphophysiological and biochemical responses of five geographically distinct populations of *Colobanthus quitensis*, originating from contrasting habitat contexts to a NaCl gradient under controlled *in vitro* conditions. We tested two explicit hypotheses. First, we hypothesized that the effects of increasing NaCl concentrations on growth-related, water-status, compatible-solute-related, antioxidant, oxidative-damage, and biochemical variables would vary among populations, resulting in population × NaCl interactions across at least a subset of the evaluated traits. Second, we hypothesized that populations with similar or partially overlapping salinity responses would exhibit distinct multivariate combinations of physiological and biochemical traits rather than a single common response profile. By integrating morphological, physiological, and biochemical variables, we aimed to characterize population-specific response profiles and determine whether salinity responses were associated with different combinations of measured traits.

## 2 Materials and methods

### 2.1 Plant material and experimental design

**Figure 1:**
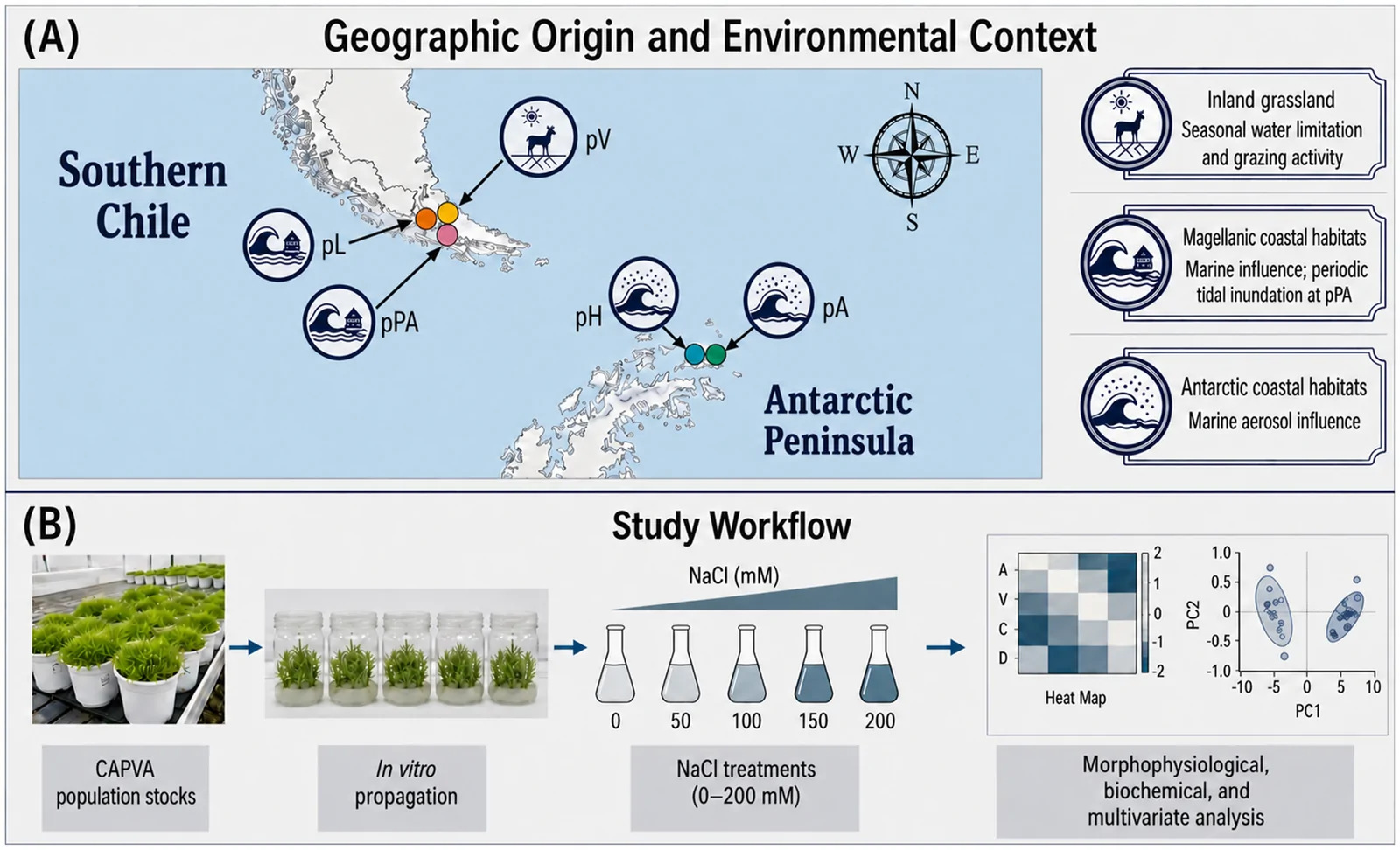
Geographic origin, general habitat context, and experimental workflow used to evaluate salinity responses in *Colobanthus quitensis*. **(A)** Locations and general habitat contexts of the five populations sources: Antarctic coastal populations exposed to marine influence (pA and pH), a Magellanic coastal salt-marsh populations subject to periodic seawater inundation (pPA), a disturbed Magellanic coastal population exposed to marine influence (pL), and an inland grassland population without direct marine influence, where seasonal water limitation and grazing activity represent potential environmental pressures (pV). These descriptors provide qualitative ecological context and were not treated as measured explanatory variables. **(B)** Plants maintained as independent stocks in the Antarctic vascular plant collection (CAPVA) were propagated under *in vitro* and exposed to 0–200 mM of NaCl for 60 days. Morphophysiological and biochemical responses were subsequently assessed and integrated using multivariate analyses to compare population-levels responses profiles.

Plant material from five geographically distinct populations of *Colobanthus quitensis* was obtained from the Antarctic Vascular Plant Collection (CAPVA), maintained at the Laboratory of Biotechnology and Environmental Studies, Universidad de Concepción, Los Ángeles Campus, Chile. The evaluated populations included Arctowski (pA), Punta Hannah (pH), Laredo (pL), La Marisma (pPA), and La Vega (pV). Their collection sites, geographic coordinates, and general habitat contexts are summarized in **Table 1** and **Figure 1**.

**Table 1:** Geographic locations, coordinates, and general habitat context of the *Colobanthus quitensis* populations included in this study.

Population	Identifier	Sampling site	General habitat context	Geographic coordinates
Arctowski	pA	King George Island, South Shetland Islands, Antarctica	Antarctic coastal ice-free habitat exposed to marine influence, including marine aerosols	62°09’44.8”S 58°28’03.9”W
Punta Hannah	pH	Livingston Island, South Shetland Islands, Antarctica	Antarctic coastal slope; marine influence and localized disturbance associated with dense seabird and marine-mammal activity	62°39’14.8”S 60°36’30.8”W
Laredo	pL	Laredo sector, North of Punta Arenas, Chile	Magellanic coastal and anthropogenically disturbed habitat; exposed to marine influence	52°58’11.6”S 70°49’33.3”W
La Marisma	pPA	Santa María Point, South of Punta Arenas, Chile	Low-elevation coastal salt marsh subject to periodic seawater inundation	53°21’59.7”S 70°58’21.9”W
La Vega	pV	INIA Kampenaike, Experimental Field (Laguna Blanca), Laguna commune, Magallanes, Chile	Inland Magellanic grassland without direct marine influence; seasonal water limitation and grazing activity as potential environmental pressures	52°41’00.0”S 70°56’00.0”W

Habitat descriptors are qualitative and are based on published regional information, collection records, and field observations. They were not measured during the present experiment and were not treated as explanatory variables.

The CAPVA stock representing each population was derived from population-level collections comprising multiple field-collected plants and/or seeds rather than from a single source individual. The original source material for pA, pH, pL, and pPA was collected in 2015, whereas that for pV was collected in 2011. In CAPVA, plants were vegetatively propagated under common-garden conditions by separating shoots from source cushions, and each population was maintained as an independent, labeled stock throughout common-garden and *in vitro* culture. To avoid continuous long-term *in vitro* maintenance, stocks were periodically renewed from common-garden material. For the salinity experiment, healthy, contamination-free plantlets from culture batches with comparable establishment dates and subculture histories were selected within each population.

The selected populations represent contrasting habitat contexts across the southern portion of the species distribution range. Antarctic populations pA and pH originate from coastal ice-free habitats exposed to marine influence; pPA originates from a low-elevation coastal salt marsh subject to periodic seawater inundation; pL represents a disturbed Magellanic coastal site exposed to marine influence; and pV originates from an inland grassland without direct marine influence where seasonal water limitation and grazing activity represent potential environmental pressures (**Table 1**; **Figure 1**). These habitat descriptors provide qualitative context for population selection. They were not measured during the present study and were not included as explanatory variables in the analyses.

Plants were propagated and maintained *in vitro* on Murashige and Skoog (MS) medium supplemented with 3% sucrose, 0.5 mg L^-^¹ 6-benzylaminopurine (BAP), and 0.25 mg L^-^¹ indole-3-acetic acid (IAA). The pH was adjusted to 5.7 prior to autoclaving, and the medium was solidified with 0.7% agar. Silver thiosulfate (10 μM) was added by sterile filtration after autoclaving to minimize ethylene-associated senescence during prolonged *in vitro* culture. Cultures were maintained in 150-mL glass flasks containing 30 mL of medium and ten plants per flask, at 18 ± 2 °C under a 16 h light/8 h dark photoperiod and a photosynthetic photon flux density of 45–50 μmol m^-^² s^-^¹. Flask positions were periodically rearranged within the growth chamber during the 60-day exposure period to reduce positional variation in light conditions.

For salinity treatments, NaCl was incorporated into the culture medium at final concentrations of 0, 50, 100, 150, and 200 mM. Prior to treatment establishment, plants were standardized to approximately 1.5 cm in total length and allocated across NaCl treatments to balance initial size and developmental status within populations. Eight independent culture flasks were established for each population × NaCl combination. Culture flasks were considered the independent experimental units, whereas plants within each flask were treated as subsamples because they shared the same culture medium and microenvironment.

Chlorosis was assessed non-destructively at the flask level. For morphophysiological and biochemical determinations, individual plants or tissue were sampled from the replicate flasks according to the material requirements of each analysis. Measurements obtained from multiple plants within the same flask were summarized to obtain a single value per experimental replicate, as appropriate for each determination. The root-to-shoot length ratio was calculated as root length divided by shoot length and was used as an indicator of relative root-to-shoot growth under salinity exposure.

The evaluated variables included chlorosis, dry biomass, root-to-shoot length ratio, tissue water content, proline concentration, total soluble sugars (TSS), malondialdehyde (MDA) content, and the activities of ascorbate peroxidase (APX), catalase (CAT), and guaiacol peroxidase (GuPX). Morphophysiological measurements were performed following procedures previously described for *C. quitensis* populations (Cuba-Díaz et al., 2025). Biochemical determinations were based on established methodologies with minor adjustments in extraction volumes and reaction scaling to accommodate the limited biomass available from *in vitro*-grown plants.

### 2.2 Biochemical analyses

Biochemical determinations were performed using frozen plant tissue collected after 60 days of salinity exposure and stored at −80 °C until analysis. Three independent biological extracts were analyzed for each population × NaCl treatment combination. All absorbance measurements were performed using a GENESYS 10S UV–Vis spectrophotometer (Thermo Scientific, USA). Because of the limited biomass of *in vitro*-grown *Colobanthus quitensis*, tissue masses and reaction volumes were adjusted for each assay. Reagent blanks were included in all spectrophotometric measurements, and technical readings were not treated as independent replicates.

For antioxidant-enzyme analyses, 150 mg of frozen tissue was ground in liquid nitrogen and extracted with 1.5 mL of 200 mM potassium phosphate buffer (pH 7.4) containing 0.1 mM EDTA and 0.1% (w/v) polyvinylpyrrolidone. Homogenates were centrifuged at 10,000 × *g* for 20 min at 4 °C, and the resulting supernatants were used as crude enzyme extracts. Total soluble protein was quantified at 595 nm using the Bradford method and bovine serum albumin as the calibration standard (Bradford, 1976). Enzyme activities were normalized to U ml^-1^ dry biomass.

Catalase (CAT) activity was determined following Frary et al. (2010), with modifications. The 2-mL reaction mixture contained 100 mM potassium phosphate buffer (pH 7.5), 10 mM H_2_O_2_, and enzyme extract containing 20 μg of total protein. H_2_O_2_ consumption was monitored at 240 nm at 10-s intervals for 5 min. Guaiacol peroxidase (GuPX) activity was measured following Mazhoudi et al. (1997) in a 2-mL reaction containing 100 mM potassium phosphate buffer (pH 7.0), 2.5 mM H_2_O_2_, 0.3 mM guaiacol, and enzyme extract containing 2 μg of total protein. The increase in absorbance at 470 nm was recorded at 10-s intervals for 10 min. Ascorbate peroxidase (APX) activity was determined following Nakano and Asada (1981) in a 2-mL reaction containing 100 mM potassium phosphate buffer (pH 7.0), 0.1 mM EDTA, 10 mM H_2_O_2_, 1 mM ascorbate, and enzyme extract containing 6 μg of total protein. Ascorbate oxidation was monitored at 290 nm at 10-s intervals for 5 min. For all enzyme assays, blanks were prepared by replacing the enzyme extract with extraction buffer.

Total soluble sugars (TSS) were quantified using the resorcinol method adapted from Roe (1934). Approximately 100 mg of frozen tissue was ground in liquid nitrogen and extracted with 1 mL of 86% (v/v) ethanol. Extracts were incubated at 60 °C for 30 min and centrifuged at 12,000 rpm for 10 min. A 100-μL aliquot of the supernatant was combined with 1.75 mL of 37% HCl, 250 μL of 1% (w/v) resorcinol, and 500 μL of deionized water. The reaction was incubated at 80 °C for 8 min, cooled to room temperature, and measured at 520 nm. TSS concentrations were calculated using a sucrose calibration curve and expressed on a fresh-weight basis. The blank contained the complete reaction mixture with the extract replaced by 86% ethanol.

Free proline content was quantified using a ninhydrin-based assay adapted from Bates et al. (1973). Approximately 200 mg of frozen tissue was extracted with 1 mL of 70% (v/v) ethanol and centrifuged at 10,000 rpm for 10 min at 4 °C. Depending on the concentration of the sample, 50–100 μL of supernatant was brought to 600 μL with 70% ethanol and mixed with 400 μL of 1% (w/v) ninhydrin prepared in acetic acid and ethanol. Samples were incubated at 95 °C for 20 min, rapidly cooled on ice, and extracted with 2 mL of toluene. Following centrifugation at 2,500 rpm for 5 min, the absorbance of the organic phase was measured at 520 nm using toluene as the blank. Proline content was calculated from a calibration curve prepared with known proline concentrations and expressed on a fresh-weight basis.

Lipid peroxidation was estimated as malondialdehyde (MDA) equivalents using a thiobarbituric acid-reactive substances assay adapted from Dhindsa et al. (1981) and previously applied to *C. quitensis* by Bertini et al. (2022) and Cuba-Díaz et al. (2025). Frozen tissue (200 mg) was ground in liquid nitrogen, homogenized in 1.5 mL of 0.1% (w/v) trichloroacetic acid, and centrifuged at 13,500 rpm for 10 min. Separate 500-μL aliquots of the supernatant were mixed with 1.25 mL of either 0.5% (w/v) thiobarbituric acid prepared in 20% trichloroacetic acid or 20% trichloroacetic acid without thiobarbituric acid. Reactions were incubated at 80 °C for 30 min and rapidly cooled on ice. Absorbance was measured at 532 and 600 nm, and nonspecific absorbance and sample turbidity were corrected using the corresponding reaction without thiobarbituric acid. MDA equivalents were calculated using a molar extinction coefficient of 155 mM^-^¹ cm^-^¹ and expressed on a fresh-weight basis.

### 2.3 Ion quantification

Concentrations of Na^+^, K^+^, Ca²^+^, and Mg²^+^ were determined for all NaCl treatments (0, 50, 100, 150, and 200 mM). For each population × NaCl combination, dry material from five plants randomly selected from the corresponding culture vessels was pooled into a single composite sample because individual plants yielded insufficient biomass for ion analysis. Samples were subjected to acid digestion with HNO_3_:H_2_O_2_, and elemental concentrations were quantified by inductively coupled plasma atomic emission spectroscopy (ICP-AES) using element-specific calibration curves, following Bojórquez-Quintal et al. (2014), with adaptations. Each extract was measured through three instrumental readings, and the mean of the three instrumental readings was used to calculate the Na^+^/K^+^, Na^+^/Ca²^+^, and Na^+^/Mg²^+^ ratios. Because each population × NaCl combination was represented by a single composite sample without independent biological replication, ion concentrations and ratios were interpreted exclusively at a descriptive level and were not included in inferential statistical or multivariate analyses.

### 2.4 Statistical and multivariate analyses

All statistical analyses were performed in R (R Core Team, 2024).

#### 2.4.1 Data preparation

The dataset integrated morphophysiological, biochemical, enzymatic, and visual-damage variables measured across populations and salinity treatments. Morphophysiological variables included dry biomass, tissue water content, and root-to-shoot length ratio. Tissue water content was calculated as the percentage of water relative to fresh weight. Plant-level measurements were averaged within each flask before analysis.

Biochemical and enzymatic variables included proline, total soluble sugars (TSS), malondialdehyde (MDA), ascorbate peroxidase (APX), guaiacol peroxidase (GuPX), and catalase (CAT). Each flask represented one biological sample, and technical readings from the same flask, when available, were averaged before analysis. Visual damage was represented by the mean chlorosis percentage of the plants evaluated within each flask. Thus, all statistical analyses were based on independent flask-level observations rather than on individual plants.

Variables included in the PCA were centered and scaled to unit variance to account for differences in measurement units. For the exploratory path analysis, physiological variables were centered within population and subsequently standardized. The classical stress tolerance index was calculated for dry biomass only, whereas the exploratory integrated performance score was scaled from 0 to 1 and oriented so that higher values represented a more favorable integrated response.

#### 2.4.2 Effects of salinity on morphophysiological and biochemical responses across *C. quitensis* populations

To evaluate the effects of population, salinity, and their interaction on each response variable, analyses were conducted using culture-flask means, with the flask treated as the independent experimental unit. NaCl treatment was modeled as a categorical predictor with five levels (0, 50, 100, 150, and 200 mM). For non-proportional response variables, two-way factorial Gaussian models were fitted including population, NaCl treatment, and their interaction as fixed effects, and model terms were evaluated using type III tests with the car package (Fox et al., 2012). Model assumptions were evaluated through residual diagnostics, Shapiro–Wilk tests for residual normality, and Levene’s tests for homogeneity of variances. When heteroscedasticity was detected, heteroscedasticity-consistent HC3 inference was used. If the HC3 covariance matrix was not estimable, generalized least-squares models with alternative variance structures were compared using AIC, and the selected model was refitted using restricted maximum likelihood for inference. Chlorosis, expressed as a flask-level proportion, was analyzed separately using beta regression with a logit link.

Partial eta-squared was calculated from the type III sums of squares of the Gaussian linear models as a measure of effect size; when HC3 or GLS provided the primary inference, these values were interpreted descriptively. To account for multiple testing across traits, omnibus *p*-values were adjusted separately for the effects of population, NaCl treatment, and their interaction using the Benjamini–Hochberg false-discovery-rate procedure. When required, pairwise comparisons were performed using estimated marginal means with the emmeans package (Lenth et al., 2019), with Tukey adjustment within each family of comparisons. Comparisons were conducted among populations within each NaCl treatment and among NaCl treatments within each population.

#### 2.4.3 Quantification of relative responses, tolerance, and physiological coordination under salinity

##### 2.4.3.1 Tolerance indices

To evaluate the magnitude and direction of salinity-induced changes, the natural log response ratio (lnRR) was calculated for each population, treatment, and response variable relative to the corresponding population-specific control. No constant was required in the primary analysis because all treatment and control means were strictly positive. To evaluate the robustness of the results, additional sensitivity analyses were conducted using constants equivalent to 0.1%, 1%, and 10% of the trait-specific median positive control mean. Positive lnRR values indicated an increase relative to the control, whereas negative values indicated a decrease. For dry biomass, tissue water content, root-to-shoot length ratio, proline concentration, total soluble sugars, and antioxidant enzyme activities, the original direction of the lnRR was retained. For the damage-related variables MDA and chlorosis, the sign was reversed so that higher oriented values consistently represented lower damage. An exploratory integrated tolerance score was then calculated using dry biomass, tissue water content, MDA, and chlorosis, representing plant performance, water status, oxidative damage, and visible injury, respectively. After orienting MDA and chlorosis so that higher values represented lower damage, responses were standardized within each trait. Dry biomass and tissue water content were averaged to obtain a performance-domain score, whereas oriented MDA and chlorosis values were averaged to obtain a damage-domain score. The two domain scores were subsequently combined with equal weighting to avoid assigning unsupported *a priori* importance to any individual trait. Higher integrated scores therefore represented a more favorable combination of biomass and tissue-water maintenance with lower MDA and chlorosis. Population-level tolerance under high salinity was summarized by averaging the scores obtained at 150 and 200 mM NaCl. The robustness of population rankings was evaluated using a leave-one-trait-out sensitivity analysis and by comparing rankings across lnRR constant scenarios using Spearman rank correlations. The integrated score was interpreted as an exploratory descriptive measure rather than as an inferential test of overall salinity tolerance. The classical stress tolerance index proposed by Fernandez (1992) was calculated separately for dry biomass only. lnRR values and exploratory tolerance scores were visualized as heatmaps using the ggplot2 package (Wickham, 2011).

##### 2.4.3.2 Principal component analysis

To explore multivariate patterns of salinity response, principal component analysis (PCA) was performed using tolerance-oriented lnRR values. Each observation corresponded to a population × non-control NaCl treatment combination, resulting in 20 observations representing five populations and four salinity treatments (50, 100, 150, and 200 mM NaCl). Control observations were excluded because their lnRR values were zero by definition. The variables included dry biomass, tissue water content, root-to-shoot length ratio, proline, total soluble sugars, MDA, APX, GuPX, CAT, and chlorosis. The PCA was conducted using the prcomp function from the stats package, with variables centered and scaled to unit variance. Variables with zero variance were excluded. When missing values occurred, they were replaced with the mean of the corresponding variable, and the number of imputed values was recorded.

PCA scores were used to visualize the distribution of population × salinity combinations in the multivariate response space. Population dispersion was represented using 95% ellipses generated for each population with the stat_ellipse() function in the ggplot2 package (Wickham, 2011). These ellipses were included only as descriptive visual summaries and were not used as inferential evidence of statistically significant separation among populations. Loadings and squared-loading contributions were extracted to identify the traits most strongly associated with PC1 and PC2, and the variance explained by each component was reported.

##### 2.4.3.3 Exploratory observed-variable path analysis

As a complementary analysis, we conducted an exploratory observed-variable path analysis using the lavaan package (Rosseel, 2012). The culture flask was considered the independent experimental unit, and plant-level measurements and technical readings obtained from the same flask were averaged before analysis. Three matched flasks were retained for each population × NaCl combination, yielding 75 observations (five populations × five NaCl treatments × three flasks). No latent variables or measurement models were fitted. Osmoprotection was represented by standardized proline concentration; antioxidant response by an equal-weighted composite calculated from z-standardized APX, CAT, and GuPX activities; damage by standardized MDA; morphological adjustment by the standardized root-to-shoot ratio; and plant performance by standardized dry biomass.

NaCl concentration was included as a standardized continuous predictor. Population was represented by four contrast-coded covariates included in each structural equation to account for differences among populations. Paths were specified *a priori* according to the proposed physiological framework linking salinity exposure, physiological responses, and plant performance. The model was fitted using robust maximum likelihood estimation (MLR). Model evaluation included robust χ², CFI, TLI, RMSEA with 95% confidence intervals, SRMR, standardized path coefficients, robust standard errors, confidence intervals, and R² values. Because all physiological variables were measured at the end of the experiment, paths were interpreted as conditional associations rather than as evidence of causal or temporal relationships.

## 3 Results

### 3.1 Population-specific phenotypic responses under salinity stress

**Figure 2:**
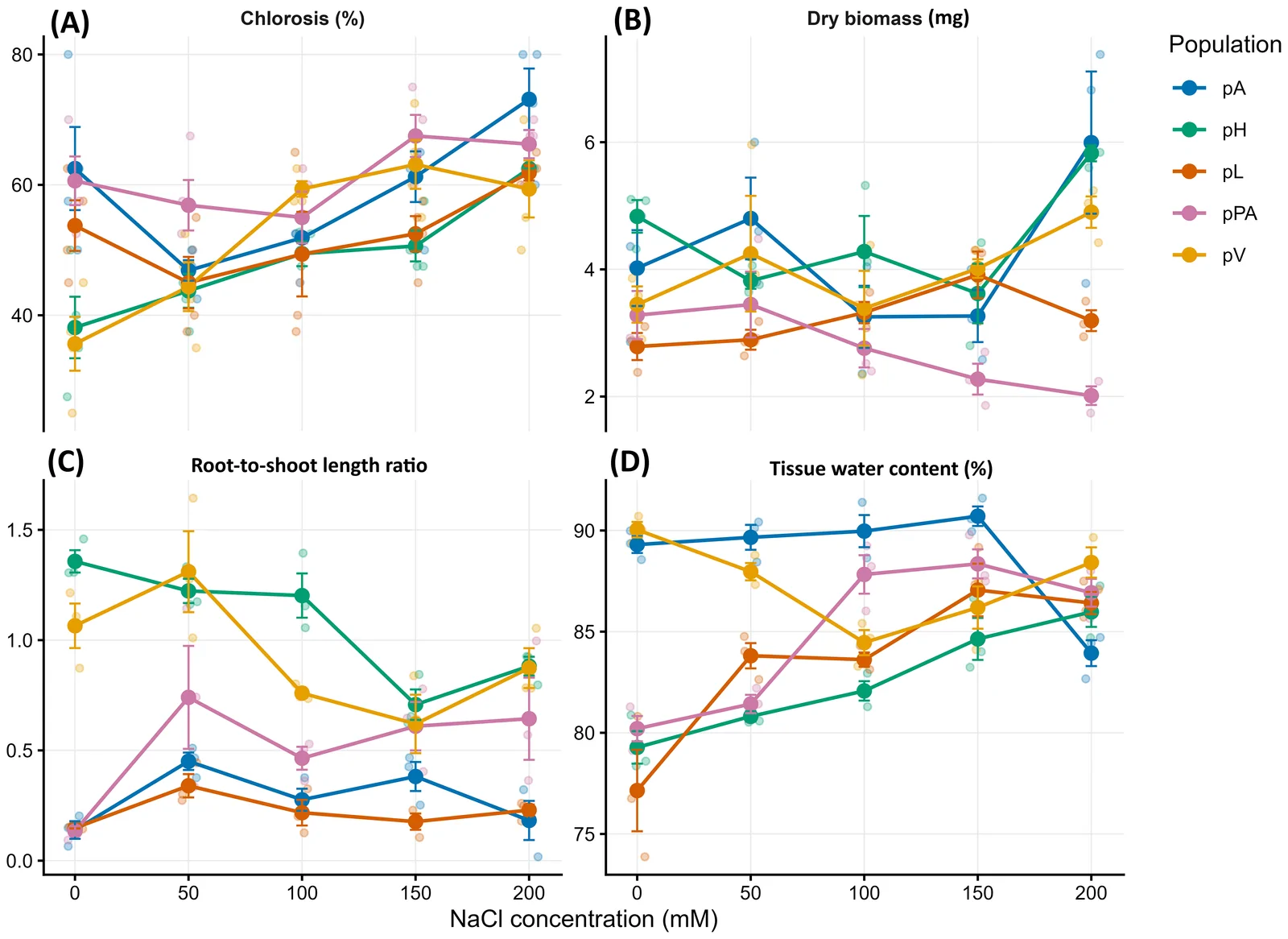
Population-specific morphophysiological responses of *Colobanthus quitensis* under increasing salinity treatments. **(A)** Chlorosis (%), **(B)** dry biomass (mg), **(C)** root-to-shoot length ratio, and **(D)** tissue water content (%) in plants exposed to 0, 50, 100, 150, and 200 mM NaCl. Large points and error bars represent means ± SE, whereas small semi-transparent points represent independent culture-flask observations. For each population × NaCl combination, chlorosis was evaluated using four independent culture flasks, whereas dry biomass, root-to-shoot length ratio, and tissue water content were evaluated using three independent culture flasks. Measurements obtained from plants within the same flask were averaged before analysis. Lines connect treatment means within each population and are included to facilitate visualization of response trajectories.

Salinity responses differed markedly among *C. quitensis* populations (**Figure 2**; **Supplementary Table 1**; **Supplementary Data Sheet 1**). Population, NaCl treatment, and their interaction significantly affected chlorosis, dry biomass, root-to-shoot length ratio, and tissue water content. Interaction effects were particularly strong for tissue water content (partial η² = 0.823), followed by root-to-shoot length ratio (partial η² = 0.576) and dry biomass (partial η² = 0.466), consistent with population-dependent response trajectories across the salinity gradient. For chlorosis, population, NaCl treatment, and their interaction were also significant (Wald χ² = 53.82, 93.92, and 61.01, respectively; all *P* < 0.001).

Chlorosis generally increased with salinity, but population trajectories differed (**Figure 2A**). Population pA showed the highest chlorosis across most treatments, increasing from 62.5% under control conditions to 73.1% at 200 mM NaCl. Population pV exhibited the largest increase, from 35.6% to 59.4%, whereas pH and pL reached approximately 62% at 200 mM. Population pPA remained comparatively stable up to 100 mM before increasing at higher salinity.

Dry biomass also showed contrasting responses (**Figure 2B**). Biomass declined by approximately 39% in pPA between the control and 200 mM NaCl, whereas it increased by approximately 49% in pA, 42% in pV, and 21% in pH. Population pL showed comparatively limited variation across treatments. Consistent with these contrasting trajectories, the effects of population, NaCl treatment, and their interaction on biomass were significant (*F* = 12.68, 3.88, and 2.72, respectively; *P* ≤ 0.008).

The root-to-shoot length ratio and tissue water content also showed population-dependent responses (**Figures 2C, D**). The root-to-shoot length ratio increased most strongly in pPA, from 0.13 to 0.64, whereas pA and pL maintained comparatively low values across treatments. Tissue water content remained generally high, but pH increased progressively with salinity, pA declined at 200 mM, and pV declined at intermediate salinity and showed higher values again at the highest concentrations. These responses were supported by significant population × NaCl interactions for root-to-shoot length ratio (*F* = 4.24, partial η² = 0.576) and tissue water content (*F* = 14.57, partial η² = 0.823; both *P* < 0.001).

### 3.2 Population-specific biochemical and oxidative stress-related responses under salinity

**Figure 3:**
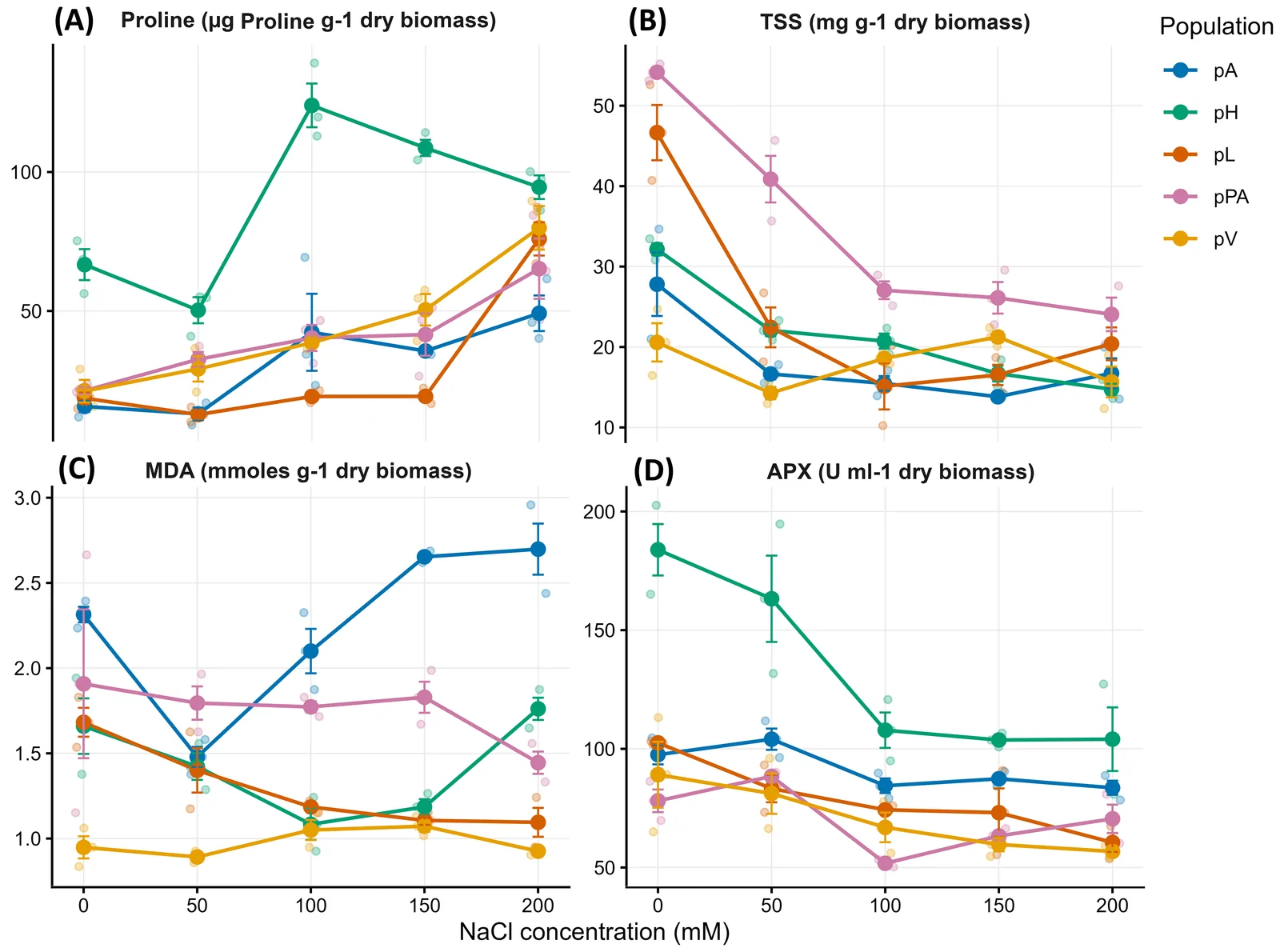
Population-specific biochemical and oxidative stress-related responses of *Colobanthus quitensis* under increasing salinity. **(A)** Proline concentration, **(B)** total soluble sugars (TSS), **(C)** malondialdehyde (MDA) content, and **(D)** ascorbate peroxidase (APX) activity in plants exposed to 0, 50, 100, 150, and 200 mM NaCl. Large points and error bars represent means ± SE, whereas small semi-transparent points represent independent culture-flask observations. For each population × NaCl combination, variables were evaluated using three independent culture flasks. Lines connect treatment means within each population and are included to facilitate visualization of response trajectories.

At the 60-day endpoint, proline concentration, total soluble sugars (TSS), ascorbate peroxidase (APX), malondialdehyde (MDA), catalase (CAT), and guaiacol peroxidase (GuPX) showed significant effects of population, NaCl treatment, and their interaction (all FDR-adjusted *P* ≤ 0.004; **Figure 3**; **Supplementary Figure 1**; **Supplementary Table 1**; **Supplementary Data Sheet 1**).

Proline accumulation differed markedly among populations (**Figure 3A**). Population pH exhibited the highest proline concentrations across treatments, increasing from 66.7 µg g^-^¹ FW under control conditions to a maximum of 124.0 µg g^-^¹ FW at 100 mM NaCl. In contrast, pL maintained comparatively low proline levels across most treatments (12.7–19.3 µg g^-^¹ FW) and increased sharply only at 200 mM NaCl, reaching 75.9 µg g^-^¹ FW. Populations pPA and pV showed progressive increases across the salinity gradient, reaching 65.2 and 79.9 µg g^-^¹ FW, respectively, at 200 mM NaCl, whereas pA exhibited intermediate but more variable responses.

Total soluble sugars (TSS) generally declined with increasing NaCl concentration (**Figure 3B**). Population pPA maintained the highest TSS concentrations across treatments despite a progressive reduction from 54.2 to 24.1 mg g^-^¹ FW between control and 200 mM NaCl. Similar declines were observed in pA, pH, and pL, whereas pV displayed comparatively smaller fluctuations across treatments. Overall, no consistent accumulation of soluble sugars was observed under increasing salinity.

Lipid peroxidation, estimated as MDA equivalents, displayed contrasting population-specific patterns (**Figure 3C**). Population pA exhibited the highest MDA concentrations at 150 and 200 mM NaCl, increasing from 2.31 µmol g^-^¹ FW in controls to 2.70 µmol g^-^¹ FW at 200 mM NaCl. In contrast, pV maintained consistently low MDA levels across treatments (0.89–1.07 µmol g^-^¹ FW). Populations pH, pL, and pPA showed intermediate or fluctuating responses, with lower MDA concentrations than pA at the two highest NaCl treatments.

Ascorbate peroxidase (APX) activity also varied among populations (**Figure 3D**). Population pH exhibited the highest APX activity under control conditions (183.9 U mg^-^¹ protein) and declined to approximately 104 U mg^-^¹ protein at 200 mM NaCl. Population pPA decreased from 88.4 U mg^-^¹ protein at 50 mM NaCl to 51.8 U mg^-^¹ protein at 100 mM NaCl, followed by higher values at 150 and 200 mM. Population pA maintained comparatively stable APX activity across treatments, whereas pL and pV showed gradual declines with increasing salinity. CAT and GuPX also showed population-dependent variation across NaCl treatments (**Supplementary Figure 1**). GuPX exhibited highly variable trajectories among populations, whereas CAT activity was generally maintained or slightly increased at 50 mM NaCl and declined in most populations at 200 mM NaCl.

Descriptive inspection of the composite samples showed that Na^+^/K^+^, Na^+^/Ca²^+^, and Na^+^/Mg²^+^ ratios generally increased with increasing NaCl concentration, although their trajectories differed among populations (**Supplementary Figure 2**). At 200 mM NaCl, pPA and pV showed lower Na^+^/K^+^ ratios than pA, pH, and pL. For Na^+^/Ca²^+^ and Na^+^/Mg²^+^, pPA displayed a non-monotonic pattern, with higher values at 100 mM, a decrease at 150 mM, and a subsequent increase at 200 mM. Because each plotted value represents the mean of three instrumental readings obtained from a single composite sample without independent biological replication, these patterns are presented solely as descriptive observations and do not support inferential comparisons among populations or treatments.

### 3.3 Exploratory multivariate analysis summarizes population-specific physiological response profiles under salinity stress

**Figure 4:**
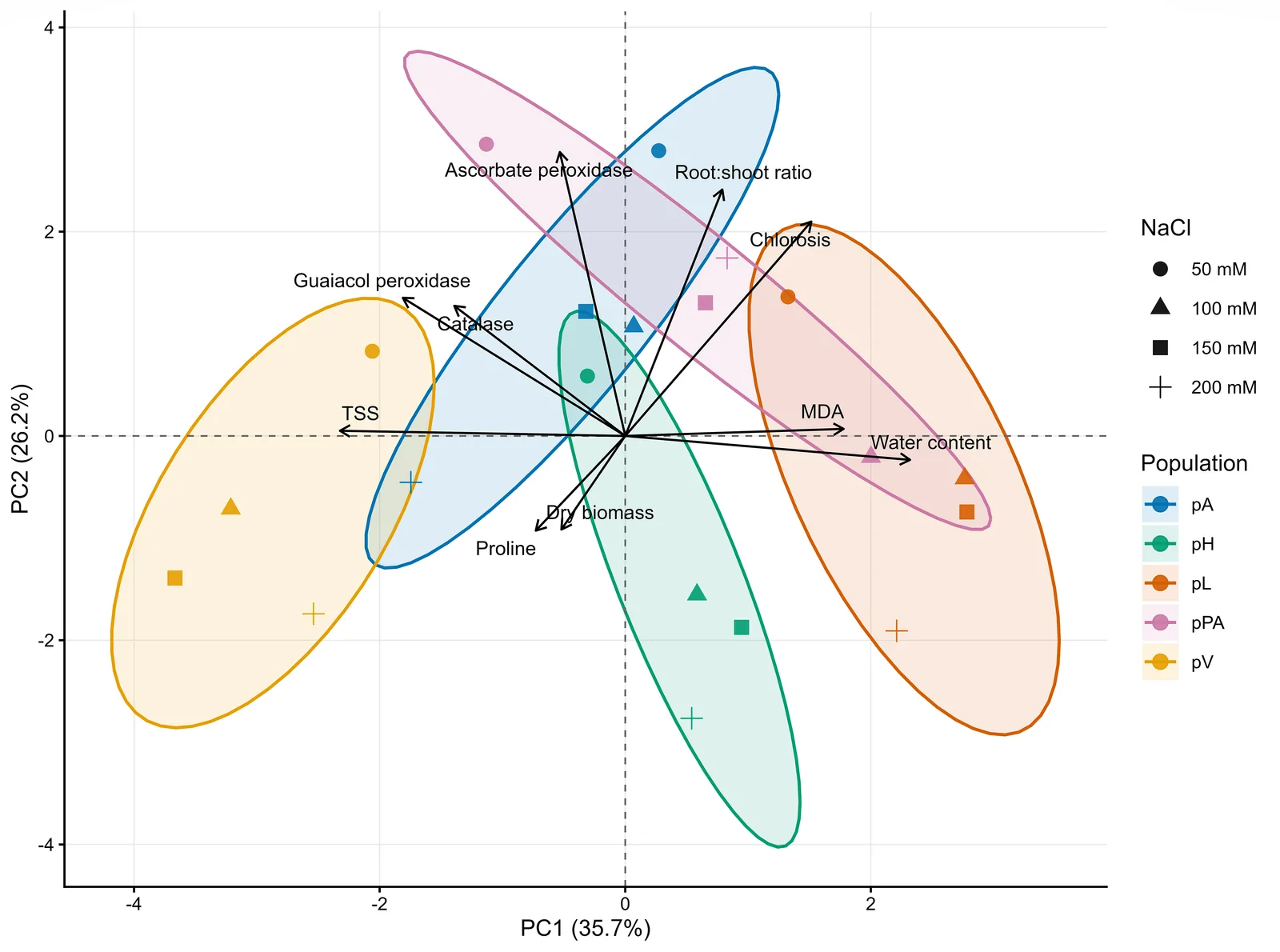
Principal component analysis of tolerance-oriented responses in *Colobanthus quitensis* populations exposed to increasing salinity. The PCA integrated morphophysiological, biochemical, oxidative-damage, and antioxidant-response variables expressed as natural log response ratios (lnRR) relative to the corresponding population-specific controls. MDA and chlorosis were reverse-oriented so that positive values consistently represented lower damage. Each point represents one population × non-control NaCl treatment combination at 50, 100, 150, or 200 mM NaCl. Symbols indicate NaCl treatments, and colors indicate populations. PC1 and PC2 explained 35.7% and 26.2% of the total variance, respectively, jointly accounting for 61.9%. Arrows represent variable loadings and indicate the direction and relative contribution of each trait to the patterns of covariation in the PCA space. Ellipses were generated for each population at the 95% level using stat_ellipse() in ggplot2 and are included only as descriptive summaries of population dispersion. They should not be interpreted as formal tests or as evidence of statistically significant separation among populations.

Principal component analysis (PCA) was used as an exploratory summary of multivariate responses to salinity (**Figure 4**; **Supplementary Figure 3**; **Supplementary Data Sheet 1**). The analysis included 20 population × non-control NaCl treatment combinations and 10 standardized lnRR response variables. No values required imputation. The first two principal components explained 61.9% of the total variance, with PC1 accounting for 35.7% and PC2 for 26.2%. PC1 mainly contrasted tissue water content (loading = 0.482), MDA (0.370), and chlorosis (0.315) with total soluble sugars (−0.483), guaiacol peroxidase (−0.376), and catalase (−0.289). PC2 was associated primarily with ascorbate peroxidase (loading = 0.578), root-to-shoot length ratio (0.501), and chlorosis (0.436). Complete PCA scores and loadings are provided in **Supplementary Data Sheet 1**, and the combined squared-loading contributions to PC1 and PC2 are summarized in **Supplementary Figure 3**.

The ordination suggested broad population-specific response tendencies. Population pV combinations were positioned mainly on the negative side of PC1, whereas pL combinations occurred predominantly on its positive side. Most pPA combinations were located on the positive side of PC2, whereas pH combinations at 100–200 mM NaCl were located toward negative PC2 values. Population pA showed comparatively broad variation across the ordination space. Because the PCA was exploratory and no formal multivariate test of group separation was performed, these patterns are interpreted as descriptive tendencies rather than as evidence of statistically significant differentiation among populations.

### 3.4 Integrated response patterns summarize population-specific trait combinations under salinity stress

**Figure 5:**
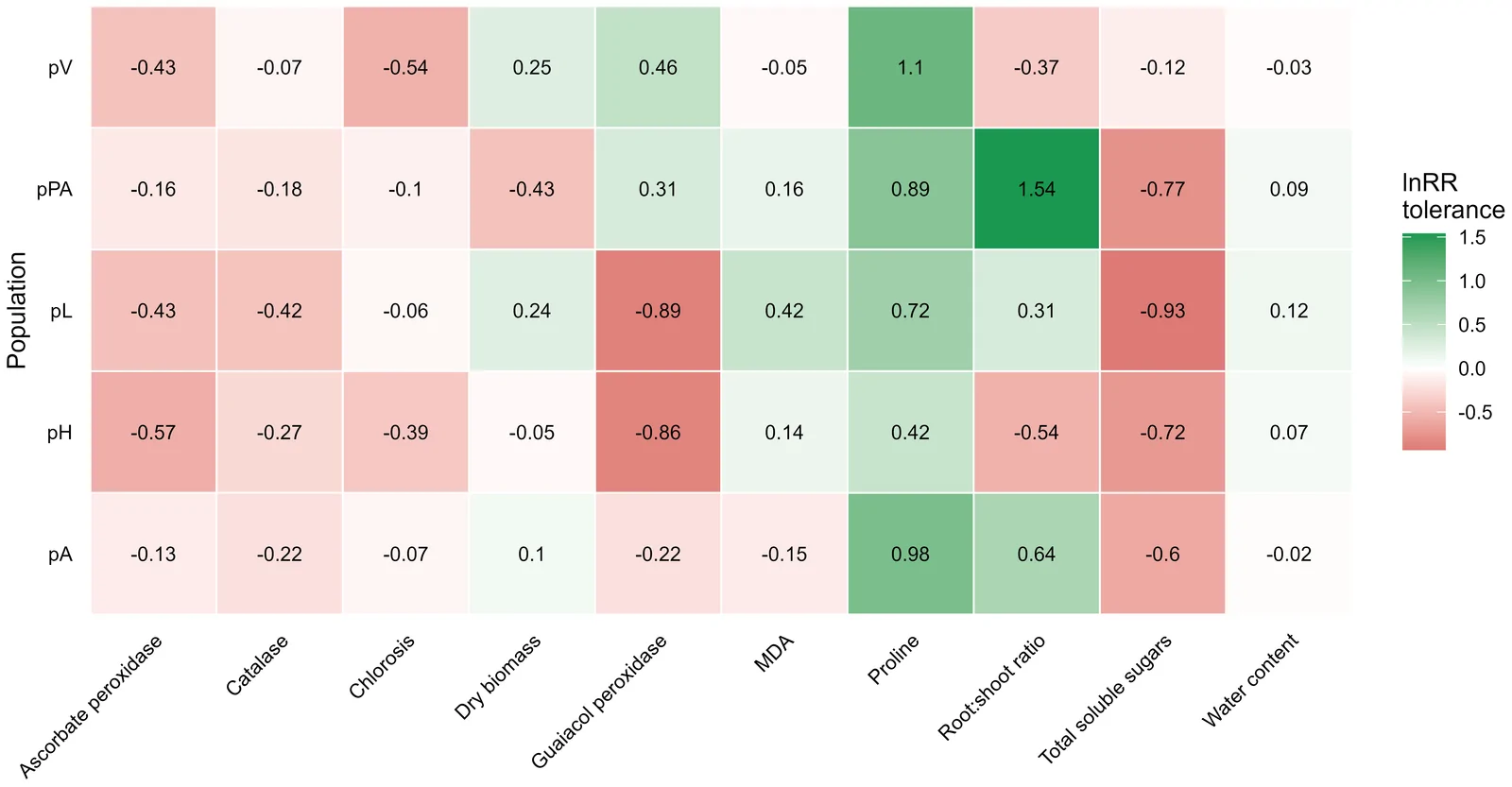
Heatmap of tolerance-oriented lnRR values for morphophysiological and biochemical traits measured in *Colobanthus quitensis* populations under the highest salinity treatment (x¯ 150–200 mM NaCl). Positive values (green) indicate trait responses associated with higher tolerance, whereas negative values (red) indicate responses associated with lower tolerance after orientation of damage-related variables.

Oriented lnRR profiles provided a descriptive summary of trait responses under high salinity, averaged across the 150 and 200 mM NaCl treatments (**Figure 5**; **Supplementary Figure 5**; **Supplementary Data Sheet 1**). Positive values represented increases in the original response variables or, for MDA and chlorosis, lower values relative to the corresponding population-specific controls after reverse orientation. The profiles indicated that populations differed in the combinations of compatible-solute-related and antioxidant responses, growth-related traits, root-to-shoot length ratio, and damage indicators expressed under salinity.

Proline showed the strongest positive response in pV (lnRR = 1.10), followed by pA (0.98), pPA (0.89), and pL (0.72). Population pPA exhibited the largest increase in root-to-shoot length ratio (lnRR = 1.54), although it also showed reductions in dry biomass (−0.43) and total soluble sugars (−0.77). Population pA combined positive responses in proline (0.98) and root-to-shoot length ratio (0.64) with a decline in total soluble sugars (−0.60). Population pV showed a positive response in guaiacol peroxidase activity (0.46), but negative oriented lnRR values for chlorosis (−0.54) and root-to-shoot length ratio (−0.37). Population pL showed positive responses for proline (0.72), the reverse-oriented MDA response (0.42), and dry biomass (0.24), together with declines in total soluble sugars (−0.93) and guaiacol peroxidase activity (−0.89). Population pH showed comparatively modest positive lnRR values for proline, the reverse-oriented MDA response, and tissue water content, but negative lnRR values for several antioxidant variables and the root-to-shoot length ratio.

The exploratory integrated response score based on dry biomass, tissue water content, chlorosis, and MDA produced the following descriptive ranking under high salinity: pV (0.84), pH (0.68), pL (0.40), pA (0.39), and pPA (0.24; **Supplementary Figure 4**; **Supplementary Data Sheet 1**). This ranking was relatively stable when each component trait was omitted in turn, with a Spearman rank correlation of 0.90 and a maximum change of one rank position. These values were interpreted as relative descriptive scores rather than as an inferential measure of overall salinity tolerance.

### 3.5 Integrated response score ranking under high salinity

**Figure 6:**
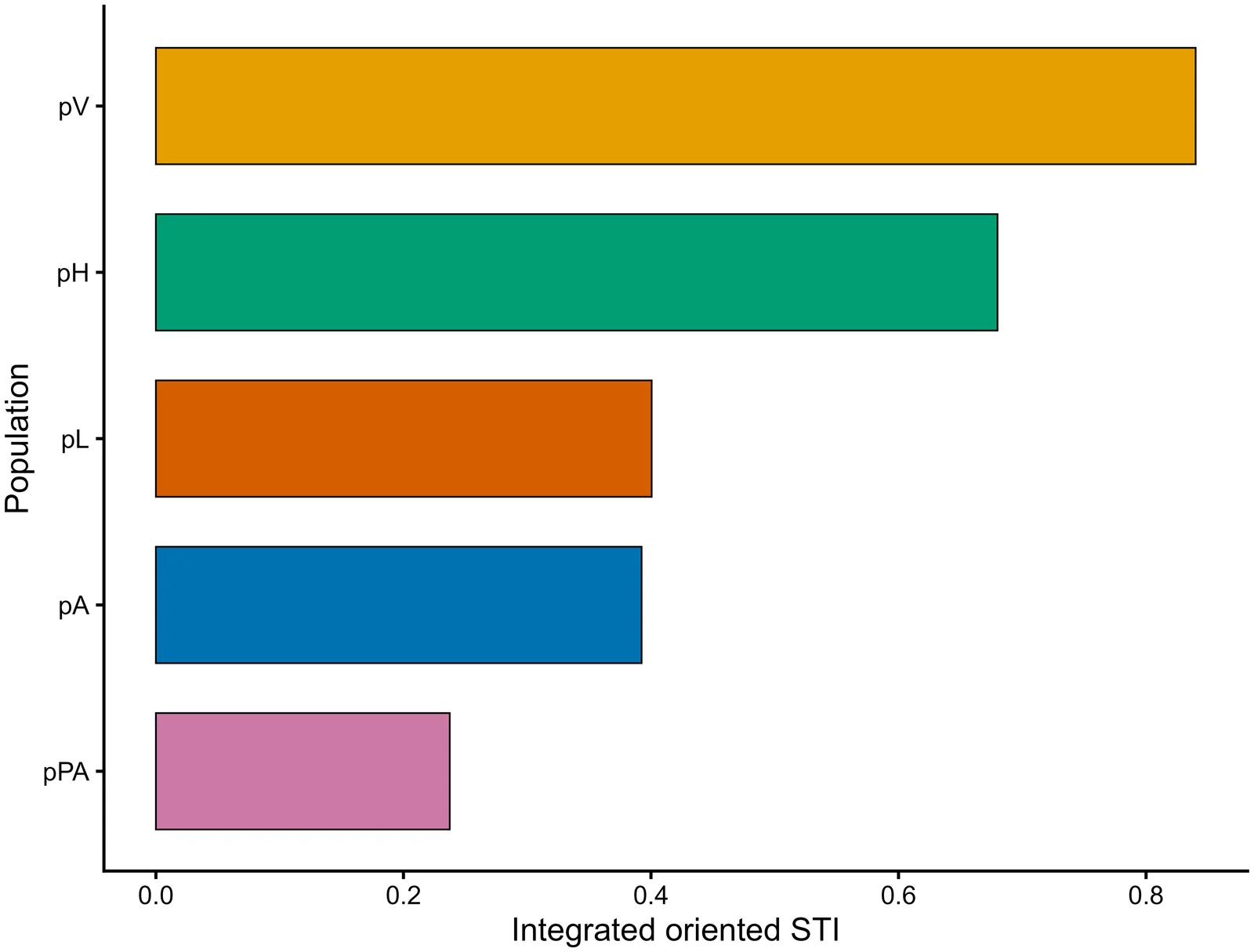
Integrated tolerance score ranking summarizing overall salinity responsiveness across *Colobanthus quitensis* populations. Rankings were generated by integrating dry biomass, tissue water content, MDA, and chlorosis responses averaged across the 150 and 200 mM NaCl treatments. MDA and chlorosis were reverse-oriented so that higher values consistently represented lower damage. All four variables contributed with equal weighting after orientation and standardization. Higher scores indicate a more favorable integrated response involving biomass and tissue-water maintenance together with lower lipid peroxidation and chlorosis. The score is interpreted as an exploratory descriptive summary rather than as an inferential or universal measure of salinity tolerance.

The exploratory integrated response score summarized the combined responses of dry biomass, tissue water content, MDA, and chlorosis under high salinity among *C. quitensis* populations, averaged across the 150 and 200 mM NaCl treatments (**Figure 6**; **Supplementary Figure 4**; **Supplementary Data Sheet 1**). Population pV showed the highest score (0.84), followed by pH (0.68). Populations pL (0.40) and pA (0.39) showed similar intermediate scores, whereas pPA showed the lowest score (0.24). These values represent a descriptive ranking of the four integrated response variables and should not be interpreted as formal statistical evidence of discrete salinity-tolerance categories among populations.

### 3.6 Exploratory observed-variable path analysis summarizes conditional associations among salinity and measured physiological responses

**Figure 7:**
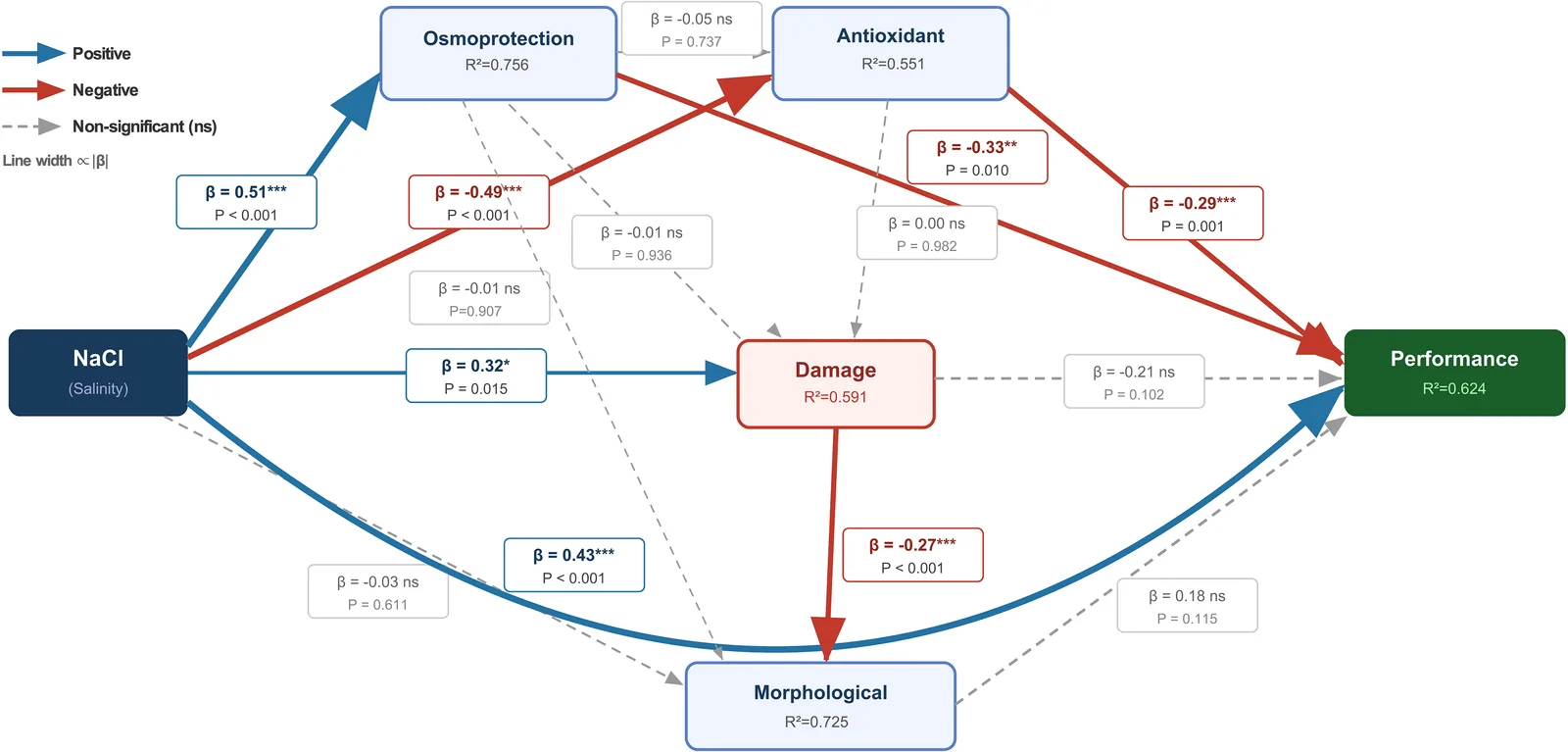
Exploratory observed-variable path model describing conditional associations among salinity, physiological responses, and plant performance. The model was fitted using 75 flask-level observations with complete and matched measurements across variables. NaCl concentration was included as a standardized continuous predictor. Population was represented by four contrast-coded covariates included in each structural equation to account for differences among populations; population-related paths are omitted from the diagram for visual clarity. Osmoprotection was represented by standardized proline concentration; antioxidant response by an equal-weighted composite calculated from z-standardized ascorbate peroxidase (APX), catalase (CAT), and guaiacol peroxidase (GuPX) activities; damage by standardized malondialdehyde (MDA); morphological adjustment by the standardized root-to-shoot length ratio; and plant performance by standardized dry biomass. The boxes therefore represent observed variables or an explicitly defined composite score; no latent variables or measurement models were fitted. These broader labels are used solely as concise graphical descriptors of the corresponding measured variables and should not be interpreted as comprehensive or latent physiological constructs. The model was estimated to use robust maximum likelihood (MLR). Arrows show standardized path coefficients (β): blue arrows indicate significant positive associations, red arrows indicate significant negative associations, and grey dashed arrows indicate nonsignificant associations (*P* ≥ 0.05). Arrow width is proportional to the absolute magnitude of the standardized coefficient. Values inside the boxes indicate the proportion of variance explained by the model (R²). Asterisks indicate statistical significance: *P* < 0.05, P < 0.01, and *P* < 0.001. Model-fit statistics were robust χ²(1) = 9.27, *P* = 0.002, CFI = 0.983, TLI = 0.091, RMSEA = 0.081, and SRMR = 0.014. Because all physiological variables were measured at the end of the experiment, the paths represent conditional associations and should not be interpreted as evidence of causal or temporal relationships. *** indicates p < 0.001, ** indicates p < 0.01, * indicates p < 0.05, and ns indicates a non-significant difference.

The observed-variable path model yielded mixed fit indices (robust χ²(1) = 9.27, *P* = 0.002; CFI = 0.983; TLI = 0.091; RMSEA = 0.081; SRMR = 0.014). Therefore, the results were interpreted cautiously as exploratory conditional associations. After accounting for population, NaCl concentration was positively associated with standardized proline concentration (β = 0.51, *P* < 0.001), standardized MDA content (β = 0.32, *P* = 0.015), and standardized dry biomass (β = 0.43, *P* < 0.001). NaCl concentration was negatively associated with the antioxidant-enzyme composite derived from standardized APX, CAT, and GuPX activities (β = −0.49, *P* < 0.001) (**Figure 7**; **Supplementary Table 2**; **Supplementary Data Sheet 2**).

Standardized MDA content was negatively associated with the standardized root-to-shoot length ratio (β = −0.27, *P* < 0.001). Proline concentration and the antioxidant-enzyme composite were also negatively associated with dry biomass (β = −0.33, *P* = 0.010 and β = −0.29, *P* < 0.001, respectively). In contrast, the associations of MDA content and root-to-shoot length ratio with dry biomass were not statistically supported (**Figure 7**; **Supplementary Table 2**). The model accounted for 62.4% of the variance in dry biomass (R² = 0.624). Robust standard errors and 95% confidence intervals for all estimated paths are provided in **Supplementary Table 2**, and the complete model output is provided in **Supplementary Data Sheet 2**. Because all physiological variables were measured at the end of the experiment, the paths represent conditional associations among the variables included in the model and should not be interpreted as causal or temporal relationships.

## 4 Discussion

### 4.1 Population-specific physiological responses under salinity stress

The present study demonstrates that salinity responses in *C. quitensis* were population dependent and involved different combinations of morphophysiological and biochemical traits rather than a uniform response pattern. Although all populations were exposed to identical *in vitro* salinity conditions, they differed in dry biomass, proline and total soluble sugar responses, antioxidant enzyme activities, MDA content, root-to-shoot length ratio, and tissue water content. These findings support the interpretation that salinity responses in *C. quitensis* arise from the integration of multiple physiological processes rather than from variation in a single measured trait, consistent with broader frameworks developed for plant responses to salinity (Busoms et al., 2023; Waheed et al., 2024).

Population-level variation was evident, although the integrated response scores did not resolve the populations into two clearly discrete tolerant and sensitive groups. The descriptive ranking nevertheless showed meaningful relative differences among populations, with higher scores in pV and pH, similar intermediate scores in pL and pA, and the lowest score in pPA (**Figure 6**; **Supplementary Figure 4**). Thus, the absence of two discrete groups should not be interpreted as equivalent responses among all populations. Instead, the populations occupied a continuum of integrated response scores and differed in the relative magnitude and combination of their measured traits. For example, some populations maintained or increased dry biomass despite comparatively high MDA levels, whereas others showed larger increases in proline concentration or root-to-shoot length ratio, or maintained comparatively high tissue water content. These results show that populations with similar or partially overlapping integrated scores may nevertheless display different physiological response profiles, consistent with previous evidence that comparable responses to stress can be associated with different combinations of physiological traits (Augstein and Carlsbecker, 2022; Schrieber et al., 2023).

The PCA, oriented lnRR profiles, and integrated response score provided complementary views of population-level variation in compatible-solute-related, antioxidant, growth-related, water-status, and damage responses (**Figures 4**, **5**, **6**; **Supplementary Figures 3**-**5**). The Antarctic populations pA and pH were positioned differently in the exploratory PCA and showed contrasting trait profiles, despite both originating from coastal habitats exposed to marine influence. Similarly, the Magellanic populations pPA and pL showed different physiological responses, although both originated from coastal habitats Taken together, these contrasts indicate that broad present-day habitat categories alone do not fully capture the physiological variation observed under salinity exposure. The qualitative habitat descriptors therefore provide an ecological context for interpreting the results rather than a formal test of environmental effects.

Previous studies have documented population-level variation in morphological and physiological traits and in responses to environmental conditions across the distribution range of *C. quitensis* (Gianoli et al., 2004; Bascuñán-Godoy et al., 2010, 2012; Cuba-Díaz et al., 2017c, 2025). This ecophysiological variation occurs against a documented background of genetic differentiation and population structure, population-level variation in genome size and possible ploidy, and a complex colonization history in the species (Cuba-Díaz et al., 2017a; b; Koc et al., 2018; Biersma et al., 2020). Together, these findings provide an independent basis for considering ecological and evolutionary history when interpreting population-specific physiological responses in *C. quitensis*.

The present results extend these observations by showing that population-level variation was expressed not only in the magnitude of individual responses but also in the combinations of measured traits under salinity. In plants, many physiological and molecular components of salinity responses are broadly shared, whereas their relative expression and coordination may vary among genotypes and populations (Augstein and Carlsbecker, 2022; Busoms et al., 2023; Schrieber et al., 2023; Waheed et al., 2024). Studies conducted specifically in *C. quitensis* have likewise documented condition- and population-dependent changes in physiological traits, stress-related gene expression, and metabolic pathways (Bascuñán-Godoy et al., 2010, 2012; Cuba-Díaz et al., 2017c). Overall, the present results demonstrate that *C. quitensis* populations express population-specific physiological response profiles under common *in vitro* salinity conditions and support a working hypothesis in which differences in the relative regulation and coordination of broadly shared stress-response processes, together with distinct environmental histories, contribute to the observed patterns.

Among the evaluated physiological variables, tissue water content, proline concentration, and total soluble sugars provided complementary information on water-status maintenance and compatible-solute-related responses across *C. quitensis* populations. Tissue water content remained generally high after 60 days of exposure, although the magnitude and direction of the response differed among populations. This pattern identifies maintenance of tissue hydration as a prominent component of the response of *C. quitensis* to prolonged NaCl exposure. Because tissue water content alone does not distinguish among osmotic adjustment, ion regulation, compatible-solute metabolism, and other processes contributing to plant water relations, the present results support hydration maintenance without assigning it to a single underlying mechanism. In plants generally, maintenance of tissue hydration under salinity may involve the coordinated contribution of several physiological processes and is also relevant to responses to drought, freezing, and dehydration (Gupta and Huang, 2014; Angon et al., 2022; Shelake et al., 2022; Cao et al., 2024). In *C. quitensis*, previous studies that dehydration and freeze–thaw exposure can substantially alter tissue water status while plants retain survival and recovery capacity (Min et al., 2024; Sulaiman et al., 2026). Together, these observations place the comparatively stable tissue water content recorded here within a broader capacity of the species to maintain physiological function under conditions that challenge plant water relations and support water-status maintenance as an important component of its response to salinity.

Proline accumulation increased with salinity in most populations, but the magnitude of this response differed markedly among them and was not consistently associated with dry biomass or the exploratory integrated response score. For example, pH exhibited the highest proline concentrations, whereas pA maintained comparatively high dry biomass under salinity despite more moderate proline levels. These patterns show that proline accumulation was an important but non-exclusive component of the population responses and, by itself, did not account for differences in biomass maintenance or integrated response scores. Previous studies in *C. quitensis* have also reported proline accumulation and changes in osmolyte-related processes under salinity, dehydration, and freezing-related conditions (Min et al., 2024; Cuba-Díaz et al., 2025; Sulaiman et al., 2026), providing species-specific context for the response observed here. Together, these findings identify proline accumulation as a recurrent component of the stress-response repertoire of *C. quitensis*, while indicating that its contribution depends on its integration with other physiological processes rather than serving as a stand-alone predictor of salinity tolerance. In other plant systems, proline accumulation has likewise been interpreted as potentially reflecting compatible-solute function, stress perception, or broader metabolic adjustment rather than acting as a direct predictor of stress tolerance (Shelake et al., 2022; Schrieber et al., 2023).

Total soluble sugars did not exhibit a generalized accumulation pattern under increasing NaCl exposure. Most populations showed stable or declining TSS concentrations, indicating that the total soluble sugar pool was not consistently increased under salinity in the populations evaluated. This pattern suggests that a generalized increase in TSS was not a dominant component of the response, while still allowing a role for carbohydrate metabolism in salinity acclimation. Previous studies in *C. quitensis* have documented the involvement of soluble sugars and related metabolic pathways in responses to low temperature, freezing, and other environmental conditions, including changes in sucrose metabolism and carbohydrate distribution among plant organs (Bascuñán-Godoy et al., 2006; Zúñiga-Feest et al., 2009; Pastorczyk et al., 2014). Metabolomic analyses have also identified carbohydrate-related changes as part of broader stress-response networks in this species (Bertini et al., 2022). Together, these studies indicate that carbohydrate metabolism contributes to the stress-response repertoire of *C. quitensis*, even when the total soluble sugar pool does not increase uniformly. Moreover, specific compounds such as sucrose, trehalose, or raffinose-family oligosaccharides may respond differently from the total soluble sugar pool and contribute in ways that are not captured by bulk TSS measurements (Shelake et al., 2022). Targeted analyses of individual carbohydrates will help determine whether such compound-specific responses contribute to the contrasting salinity profiles observed among populations.

The maintenance of tissue water content despite contrasting proline and TSS responses indicates that these endpoint variables were not tightly coupled across populations. This pattern supports the interpretation that hydration maintenance in *C. quitensis* depended on a broader combination of physiological and metabolic responses rather than on the magnitude of proline or total soluble sugar accumulation alone. In plants exposed to salinity, maintenance of tissue hydration may involve coordinated changes in solute composition, cellular water relations, ion balance, and other processes not directly measured in this study (Gupta and Huang, 2014; Liu et al., 2024; Martins et al., 2024). Previous studies in *C. quitensis* have documented changes in osmolyte metabolism and tissue water status during dehydration and freeze–thaw response (Min et al., 2024; Sulaiman et al., 2026), while studies in other plant systems have identified physiological overlap among salinity, drought, and dehydration responses (Angon et al., 2022; Cao et al., 2024). Together, this evidence supports the hypothesis that different combinations of water-status regulation, compatible-solute metabolism, and ion-related processes may contribute to the population-specific responses observed under salinity.

Collectively, the present results show that tissue water content was generally maintained under prolonged NaCl exposure, whereas proline and TSS responses varied among *C. quitensis* populations. This contrast indicates that hydration maintenance was achieved through population-specific combinations of compatible-solute-related and other physiological responses rather than through a uniform osmotic profile. Together, these findings identify water-status maintenance as an important component of the response of *C. quitensis* to salinity and support the existence of different physiological configurations among populations. Future studies integrating direct measurements of water and osmotic potential with targeted osmolyte and replicated ion analyses will help clarify the mechanisms underlying these contrasting response profiles.

### 4.2 Population responses to salinity were not consistently aligned with broad present-day habitat categories

One of the most notable results of the present study was the response of the inland population pV. This population showed comparatively low MDA concentrations and the highest exploratory integrated response score under high salinity, averaged across the 150 and 200 mM NaCl treatments, despite originating from a habitat without direct marine influence (**Figure 6**; **Supplementary Figure 4**). Although pV did not exhibit the most favorable response for every measured trait, its combination of low MDA content and the highest integrated response score demonstrates that the absence of direct marine influence was not accompanied by a uniformly weak response to NaCl exposure. A previous study in *C. quitensis*, similarly documented substantial salinity tolerance among populations originating from environmentally contrasting habitats (Cuba-Díaz et al., 2025). Together, these observations indicate that broad present-day habitat categories alone do not consistently account for the population-level responses observed under controlled salinity exposure.

Previous studies in *C. quitensis* have documented substantial population-level variation in responses to salinity and other environmental conditions (Gianoli et al., 2004; Bascuñán-Godoy et al., 2010, 2012; Cuba-Díaz et al., 2017a; Ontivero et al., 2024; Cuba-Díaz et al., 2025). The present study extends these observations by showing that the ranking of the evaluated populations did not follow a simple coastal-to-inland pattern. Population pV showed the highest integrated response score, followed by the Antarctic coastal population pH; pL and pA showed similar intermediate scores, whereas the coastal population pPA showed the lowest score. As an exploratory synthesis of four response variables, this score is most appropriately interpreted as a comparative continuum rather than as a set of discrete tolerance categories. Within this continuum, the ranking provides clear evidence that the responses expressed under common *in vitro* conditions were not ordered according to the populations’ broad degree of present-day marine influence.

In plants generally, salinity and dehydration can involve overlapping physiological and molecular processes, including changes in water relations, compatible-solute metabolism, redox regulation, and stress-signaling networks (Shelake et al., 2022; Zhu et al., 2025; Kanwal et al., 2026). Studies conducted specifically in *C. quitensis* further have also documented changes in tissue water status, osmolyte-related metabolism, and physiological function during dehydration and freeze–thaw exposure (Min et al., 2024; Sulaiman et al., 2026). In other plant species, experimental evidence has shown that responses to drought or dehydration can sometimes influence responses to salinity through partially shared physiological processes (Pushpavalli et al., 2020; Angon et al., 2022; Cao et al., 2024). This evidence provides a biologically plausible framework for proposing that physiological capacities expressed under other water-related environmental challenges may also contribute to salinity responses in *C. quitensis*. Although cross-tolerance was not evaluated directly here, the contrasting population profiles are compatible with this hypothesis and provide a basis for its future experimental examination.

The comparatively high integrated response score of pV is particularly relevant in this context because its source habitat is an inland grassland qualitatively described as lacking direct marine influence and experiencing water limitation and grazing disturbance. These characteristics provide useful ecological context for interpreting the response of this population and support the hypothesis that physiological traits associated with other environmental challenges may also contribute to its response to salinity. Several non-exclusive influences—including recurrent water limitation, grazing disturbance, local microenvironmental conditions, inherited environmental effects, long-term acclimation, and genetic differentiation—could contribute to the physiological profile expressed by pV (Cuba-Díaz et al., 2017b; Koc et al., 2018). Relationships among responses to different abiotic challenges have been documented in other plant systems (Pushpavalli et al., 2020; Cao et al., 2024), making this a biologically grounded explanation and a relevant direction for comparative experiments involving salinity, dehydration, and other environmental factors.

Comparisons among the other *C. quitensis* populations reinforce this interpretation. The Antarctic populations pA and pH, both associated with coastal habitats influenced by marine aerosols, showed different trait profiles and positions in the exploratory PCA (**Figures 4**, **5**). Likewise, the Magellanic populations pPA and pL also differed in their physiological responses despite both originating from coastal environments. Although the habitat descriptors were qualitative and were not formally modelled as environmental predictors, the repeated absence of a simple correspondence between broad habitat categories and physiological profiles supports the conclusion that present-day marine influence alone is insufficient to explain the observed variation. Instead, the results identify local environmental conditions, population structure, inherited environmental effects, demographic trajectories, and longer-term ecological and evolutionary histories as complementary and testable influences on salinity responses in the species (Cuba-Díaz et al., 2017b; c; Koc et al., 2018; Biersma et al., 2020). Integrating physiological experiments with environmental monitoring and population-genomic information will allow these influences to be evaluated directly and will help explain how contrasting response profiles have arisen across the distribution of *C. quitensis*.

### 4.3 Population-specific antioxidant responses and their associations with oxidative damage and dry biomass

In plants, salinity exposure can promote the accumulation of reactive oxygen species (ROS), potentially affecting membrane integrity, cellular metabolism, and photosynthetic function. Enzymatic antioxidant systems are therefore widely recognized as one component of plant responses to salinity (Waheed et al., 2024). In the present study, APX, CAT, and GuPX activities in *C. quitensis* showed significant effects of population, NaCl treatment, and their interaction, but their response patterns were not consistently aligned with oxidative damage or with the exploratory integrated response score. This lack of parallel variation indicates that antioxidant-enzyme activity, lipid peroxidation, and the integrated response score represented related but distinct dimensions of the population responses to salinity. This observation is consistent with previous studies in *C. quitensis* showing that responses to environmental stress cannot always be explained by variation in a single protective trait (Bertini et al., 2022; Cuba-Díaz et al., 2025).

At the 60-day endpoint, the three antioxidant enzymes showed different response patterns across populations. APX activity declined with increasing NaCl concentration in several populations, whereas CAT activity was generally maintained or slightly increased at low NaCl concentrations and tended to decline at intermediate or high concentrations. GuPX showed greater variation in both the direction and magnitude of its responses among populations (**Figure 3**; **Supplementary Figure 1**). These endpoint measurements show that antioxidant-enzyme responses were population specific and were not uniformly sustained at the highest NaCl concentrations. Because antioxidant activity is dynamic, early induction, transient activation, subsequent acclimation, enzyme turnover, and changes in non-enzymatic antioxidant systems may also have contributed to the responses expressed during the experiment. Thus, the 60-day measurements capture the established antioxidant status after prolonged exposure while also pointing to temporal variation as a relevant explanation for the contrasting enzyme profiles.

MDA content also varied among *C. quitensis* populations, but its pattern did not consistently mirror the activities of the measured antioxidant enzymes. Population pA showed the highest MDA concentrations at 150 and 200 mM NaCl, whereas pV maintained comparatively low values across the salinity gradient. Nevertheless, populations with high exploratory integrated response scores did not consistently show the highest APX, CAT, or GuPX activities, and higher enzyme activities were not uniformly associated with lower MDA content. These results indicate that antioxidant-enzyme activity and lipid peroxidation were not related through a simple inverse pattern at the 60-day endpoint. This lack of direct correspondence is physiologically plausible because MDA accumulation may also be influenced by non-enzymatic antioxidant compounds, membrane composition, lipid turnover, and membrane-repair processes. Accordingly, the observed MDA patterns likely reflect the balance among several oxidative and cellular processes rather than antioxidant-enzyme activity alone. In other plant systems, oxidative damage indicators and the maintenance of growth or physiological function have not always varied in parallel under stress, supporting the interpretation of antioxidant and oxidative-damage responses within a broader physiological context rather than as isolated predictors of stress tolerance (Schrieber et al., 2023; Waheed et al., 2024).

The exploratory observed-variable path analysis provided a complementary summary of conditional associations among the endpoint variables after accounting for population. NaCl concentration was positively associated with proline concentration (β = 0.51, *P* < 0.001), MDA content (β = 0.32, *P* = 0.015), and dry biomass (β = 0.43, *P* < 0.001), and negatively associated with the antioxidant-enzyme composite derived from standardized APX, CAT, and GuPX activities (β = −0.49, *P* < 0.001). MDA content was negatively associated with the root-to-shoot length ratio (β = −0.27, *P* < 0.001). Proline concentration and the antioxidant-enzyme composite were negatively associated with dry biomass (β = −0.33, *P* = 0.010 and β = −0.29, *P* = 0.001, respectively), whereas the associations of MDA content (β = −0.21, *P* = 0.102) and root-to-shoot length ratio (β = 0.18, *P* = 0.115) with dry biomass were not statistically supported. Likewise, the paths linking proline concentration or the antioxidant-enzyme composite with MDA content were not statistically supported (**Figure 7**; **Supplementary Table 2**; **Supplementary Data Sheet 2**).

Taken together, these conditional associations indicate that proline accumulation, antioxidant-enzyme activity, lipid peroxidation, morphological response, and dry biomass did not form a simple linear physiological sequence at the experimental endpoint. The negative associations of proline concentration and the antioxidant-enzyme composite with dry biomass are best interpreted as components of this multivariate response rather than as evidence that proline or antioxidant activity reduced growth. Higher values may have occurred in experimental units expressing a stronger physiological response to salinity, or the measured variables may have covaried with additional processes not represented in the model. Because all variables were measured at the same endpoint, the analysis does not establish temporal direction among these associations. Likewise, the antioxidant-enzyme composite summarizes APX, CAT, and GuPX activity rather than the full antioxidant capacity of the plants. Nevertheless, the model accounted for 62.4% of the variance in dry biomass and identified a structured set of population-adjusted associations that complements the univariate and integrated analyses. Given the mixed fit indices, these relationships are interpreted as exploratory conditional associations rather than as causal pathways.

Overall, the results show that antioxidant responses in *C. quitensis* were strongly population specific and only partially aligned with MDA content, dry biomass, and the exploratory integrated response score. Rather than diminishing the relevance of antioxidant regulation, this pattern places APX, CAT, and GuPX within a broader physiological network involving water relations, compatible-solute metabolism, growth, oxidative damage, and cellular maintenance (Augstein and Carlsbecker, 2022; Bertini et al., 2022; Busoms et al., 2023; Waheed et al., 2024). The contrasting enzyme trajectories and the conditional relationships identified by the path analysis therefore support the interpretation that oxidative responses contribute differently to the salinity-response profiles of individual *C. quitensis* populations. Time-course experiments incorporating earlier sampling points, non-enzymatic antioxidants, additional redox markers, and membrane-related variables will help clarify the timing and relative contribution of these processes across populations.

### 4.4 Environmental history and physiological diversification in *Colobanthus quitensis*

The controlled common-environment *in vitro* design allowed population responses to NaCl to be compared under standardized conditions, although it does not reproduce the full complexity of the source habitats. The physiological and biochemical measurements represent the 60-day endpoint, and the ion ratios are interpreted descriptively because they were obtained from pooled material without independent biological replication. Site-specific environmental variables and molecular or genomic responses were not evaluated within this experiment. These boundaries do not diminish the significant population × NaCl effects or the contrasting multivariate response profiles identified here, but they define the level at which ecological, evolutionary, and mechanistic explanations are advanced—as biologically grounded hypotheses for future testing.

Previous studies have documented substantial population-level variation in morphological, physiological, and stress-related traits across the distribution range of *C. quitensis* (Gianoli et al., 2004; Bascuñán-Godoy et al., 2010, 2012; Cuba-Díaz et al., 2017c, 2025). The present results extend these observations by showing that, under common *in vitro* salinity conditions, population differed not only in the magnitude of individual traits but also in the combinations of growth-related, water-status, compatible-solute-related, antioxidant, and oxidative-damage responses. Because all populations were evaluated under the same experimental conditions, these patterns reveal persistent population-level variation rather than immediate effects of the source environments during the experiment. Such variation is consistent with the hypothesis that long-term exposure to different combinations of abiotic and biotic pressures has contributed to physiological diversification within the species.

The contrasting responses of the Antarctic populations pA and pH illustrate this complexity. Although both originate from coastal habitats influenced by marine aerosol, they showed contrasting physiological profiles and occupied different regions of the exploratory multivariate ordination. Similarly, the Magellanic populations pPA and pL differed markedly in their response profiles despite both being associated with coastal environments. Together with the comparatively high integrated response score of the inland population pV, these patterns indicate that broad habitat categories such as coastal, Antarctic, or inland do not fully capture the population-level variation observed under salinity exposure. Importantly, these ecological contrasts occur against a documented background of morphophenotypic and genetic differentiation, population subdivision and limited gene flow, population-level variation in genome size and possible ploidy, and a complex late-Pleistocene colonization history in *C. quitensis* (Cuba-Díaz et al., 2017b; c; Koc et al., 2018; Biersma et al., 2020). Local microenvironmental conditions, disturbance regimes, demographic trajectories, inherited environmental effects, and genetic differentiation may therefore represent complementary and non-exclusive influences on the physiological profiles expressed by each population. The present results therefore support environmental history as a relevant framework for interpreting population variation in *C. quitensis*, while also highlighting the need to identify which components of that history are most influential.

Rather than supporting a single salinity-tolerant phenotype, our results indicate that *C. quitensis* populations express multiple physiological response profiles under a common salinity treatment. This diversity may be particularly relevant in a species distributed across highly heterogeneous environments, where different combinations of traits could contribute to the maintenance of physiological function under changing conditions. Whether these profiles arise primarily from differential regulation of broadly shared stress-response pathways, from population-specific molecular components, or from a combination of both remains an important question. Transcriptomic and metabolomic studies in *C. quitensis* have shown that environmental conditions modulate the expression of stress-related genes and metabolic pathways involved in acclimation and, when post-stress phases have been examined, in recovery processes (Cho et al., 2018; Bertini et al., 2022; Min et al., 2024). Together with the physiological patterns reported here, this evidence supports a working model in which populations share a broadly conserved framework for stress perception and response but differ in the magnitude, timing, and coordination of its component processes. Integrating detailed environmental characterization with population genomics, physiology, transcriptomics, metabolomics, and functional genomics will help determine how ecological and evolutionary histories contributes to physiological diversification across *C. quitensis* populations.

## 5 Conclusions

This study demonstrates that five *Colobanthus quitensis* populations exposed to the same *in vitro* NaCl gradient expressed strongly population-dependent responses in dry biomass, root-to-shoot length ratio, tissue water status, compatible-solute-related variables, antioxidant enzyme activities, and oxidative damage. Rather than exhibiting a uniform salinity-response profile, the populations differed in both the magnitude and combination of these traits. Proline accumulation and maintenance of tissue water content were recurrent components of the response, whereas antioxidant enzyme activities and MDA content showed contrasting population-specific trajectories and were not consistently aligned with the exploratory integrated response score. Together, the univariate, multivariate, integrated-score, and observed-variable path analyses support the interpretation that salinity responses in *C. quitensis* involve multiple, partially independent physiological dimensions rather than a single dominant protective trait.

Extending previous evidence of population-specific salinity tolerance in *C. quitensis*, our results show that population-level variation also encompasses distinct multivariate physiological configurations under common experimental conditions. Contrasting profiles among populations originating from broadly similar habitats, together with the comparatively high integrated response score of the inland population pV, indicate that present-day marine influence alone does not fully account for the observed variation. These patterns support the hypothesis that population-specific ecological and evolutionary histories may contribute to the diversification of salinity responses through differences in the relative expression and coordination of broadly shared physiological processes. Future integration of physiology with detailed environmental characterization, population genomics, transcriptomics, metabolomics, and functional analyses will help test this hypothesis and clarify the biological basis of population-level salinity tolerance across the heterogeneous distribution of *C. quitensis*.

## Data Availability

The raw data supporting the conclusions of this article will be made available by the authors, without undue reservation.
