# Supplementary material for: Population-specific physiological response profiles characterize salinity tolerance in the Antarctic extremophile Colobanthus quitensis
Source: Front Plant Sci. 2026 Aug 10;17:1909187. doi: 10.3389/fpls.2026.1909187 (PMC13500301; doi:10.3389/fpls.2026.1909187)
Supplement: Supplementary file 1 [file SupplementaryFile1.zip › Supplementary Material/Supplementary Figure S1.DOCX]

Supplementary Material

**Supplementary Figure S1:** Population-specific antioxidant enzyme responses of *Colobanthus quitensis* under increasing salinity treatments. (A) Guaiacol peroxidase (GuPX) activity and (B) catalase (CAT) activity in plants exposed to NaCl concentrations ranging from 0 to 200 mM. Values represent means ± SE for each population. Large points and error bars represent means ± SE, whereas small semi-transparent points represent independent culture-flask observations. Lines connect treatment means within each population.
