# Supplementary material for: Population-specific physiological response profiles characterize salinity tolerance in the Antarctic extremophile Colobanthus quitensis
Source: Front Plant Sci. 2026 Aug 10;17:1909187. doi: 10.3389/fpls.2026.1909187 (PMC13500301; doi:10.3389/fpls.2026.1909187)
Supplement: Supplementary file 1 [file SupplementaryFile1.zip › Supplementary Material/Supplementary Figure S2.DOCX]

Supplementary Material

**Supplementary Figure S2:** Descriptive ionic ratios in *Colobanthus quitensis* under increasing salinity. (A) Na⁺/K⁺, (B) Na⁺/Ca²⁺, and (C) Na⁺/Mg²⁺ ratios in five populations exposed to 0, 50, 100, 150, and 200 mM NaCl. Each plotted ratio was calculated from ion concentrations obtained as the mean of three instrumental readings of a single extract derived from one composite sample prepared by pooling dry material from five plants randomly selected for each population × NaCl combination. The instrumental readings did not constitute independent biological replicates. Lines are included only to facilitate visualization of the descriptive patterns; no inferential statistical comparisons were performed.
