# Supplementary material for: Population-specific physiological response profiles characterize salinity tolerance in the Antarctic extremophile Colobanthus quitensis
Source: Front Plant Sci. 2026 Aug 10;17:1909187. doi: 10.3389/fpls.2026.1909187 (PMC13500301; doi:10.3389/fpls.2026.1909187)
Supplement: Supplementary file 1 [file SupplementaryFile1.zip › Supplementary Material/Supplementary Figure S3.DOCX]

Supplementary Material

**Supplementary Figure S3:** Combined squared-loading contributions of the physiological and biochemical variables to the first two principal components (PC1 + PC2) of the exploratory PCA. Bars represent the sum of the squared loadings for PC1 and PC2 and describe the relative contribution of each variable to the variation represented by the first two axes.
