# Supplementary material for: Population-specific physiological response profiles characterize salinity tolerance in the Antarctic extremophile Colobanthus quitensis
Source: Front Plant Sci. 2026 Aug 10;17:1909187. doi: 10.3389/fpls.2026.1909187 (PMC13500301; doi:10.3389/fpls.2026.1909187)
Supplement: Supplementary file 1 [file SupplementaryFile1.zip › Supplementary Material/Supplementary Figure S4.DOCX]

Supplementary Material

**Supplementary Figure S4:** Stress-tolerance responses of five populations evaluated using four outcome variables. The heatmap shows oriented values rescaled from 0 to 1 for dry biomass, water content, MDA, and chlorosis. These variables were selected because they represent direct outcomes of stress across two complementary domains: performance, represented by dry biomass and water content, and damage, represented by MDA and chlorosis. MDA and chlorosis values were inverted so that, for all four variables, values close to 1 indicate greater tolerance, whereas values close to 0 indicate lower tolerance.
