# Supplementary material for: Population-specific physiological response profiles characterize salinity tolerance in the Antarctic extremophile Colobanthus quitensis
Source: Front Plant Sci. 2026 Aug 10;17:1909187. doi: 10.3389/fpls.2026.1909187 (PMC13500301; doi:10.3389/fpls.2026.1909187)
Supplement: Supplementary file 1 [file SupplementaryFile1.zip › Supplementary Material/Supplementary Figure S5.DOCX]

Supplementary Material

**Supplementary Figure S5:** Treatment-specific tolerance-oriented lnRR heatmaps showing the direction and magnitude of morphophysiological and biochemical responses across populations at 50, 100, 150, and 200 mM NaCl. Positive values indicate increases in favorable traits or decreases in damage-related traits relative to the corresponding population-specific controls, whereas negative values indicate the converse.
