# Supplementary material for: Population-specific physiological response profiles characterize salinity tolerance in the Antarctic extremophile Colobanthus quitensis
Source: Front Plant Sci. 2026 Aug 10;17:1909187. doi: 10.3389/fpls.2026.1909187 (PMC13500301; doi:10.3389/fpls.2026.1909187)
Supplement: Supplementary file 1 [file SupplementaryFile1.zip › Supplementary Material/Supplementary Table S1.DOCX]

Supplementary Material

**Supplementary Table 1:** Effects of population, NaCl treatment, and their interaction on morphophysiological and biochemical responses of *Colobanthus quitensis*. Gaussian response variables were analyzed using two-way factorial linear models, with population, NaCl treatment, and their interaction included as fixed effects. Chlorosis was analyzed using beta regression with a logit link, and model terms were evaluated using Wald chi-square tests. Partial eta-squared (partial η²) is reported as a measure of effect size for Gaussian response variables. Effect sizes are not reported for chlorosis because it was analyzed using beta regression. Values in bold may be used to indicate statistically significant effects at *p* < 0.05.

| ***Trait*** | ***Effect*** | ***Test statistic*** | ***p-value*** | ***Partial η²*** |
| --- | --- | --- | --- | --- |
| Dry biomass | Population | *F=12.68* | <0.001 | 0.504 |
|  | NaCl | *F=3.88* | 0.008 | 0.237 |
|  | Population × NaCl | *F=2.72* | 0.004 | 0.466 |
| Water content | Population | *F=52.44* | <0.001 | 0.808 |
|  | NaCl | *F=20.09* | <0.001 | 0.616 |
|  | Population × NaCl | *F=14.57* | <0.001 | 0.823 |
| Root-to-shoot length ratio | Population | *F=81.18* | <0.001 | 0.867 |
|  | NaCl | *F=8.13* | <0.001 | 0.394 |
|  | Population × NaCl | *F=4.24* | <0.001 | 0.576 |
| Chlorosis | Population | *χ²=53.82* | <0.001 | — |
|  | NaCl | *χ²=93.92* | <0.001 | — |
|  | Population × NaCl | *χ²=61.01* | <0.001 | — |
| Proline | Population | *F=96.34* | <0.001 | 0.885 |
|  | NaCl | *F=58.35* | <0.001 | 0.824 |
|  | Population × NaCl | *F=6.89* | <0.001 | 0.688 |
| Total soluble sugars | Population | *F=68.04* | <0.001 | 0.845 |
|  | NaCl | *F=84.36* | <0.001 | 0.871 |
|  | Population × NaCl | *F=9.80* | <0.001 | 0.758 |
| Malondialdehyde | Population | *F=84.41* | <0.001 | 0.871 |
|  | NaCl | *F=4.33* | 0.004 | 0.301 |
|  | Population × NaCl | *F=6.32* | <0.001 | 0.672 |
| Ascorbate peroxidase | Population | *F=65.73* | <0.001 | 0.840 |
|  | NaCl | *F=28.03* | <0.001 | 0.692 |
|  | Population × NaCl | *F=3.61* | <0.001 | 0.536 |
| Catalase | Population | *F=51.12* | <0.001 | 0.804 |
|  | NaCl | *F=44.68* | <0.001 | 0.781 |
|  | Population × NaCl | *F=5.64* | <0.001 | 0.644 |
| Guaiacol peroxidase | Population | *F=24.87* | <0.001 | 0.665 |
|  | NaCl | *F=18.03* | <0.001 | 0.591 |
|  | Population × NaCl | *F=8.94* | <0.001 | 0.741 |
