# Supplementary material for: Population-specific physiological response profiles characterize salinity tolerance in the Antarctic extremophile Colobanthus quitensis
Source: Front Plant Sci. 2026 Aug 10;17:1909187. doi: 10.3389/fpls.2026.1909187 (PMC13500301; doi:10.3389/fpls.2026.1909187)
Supplement: Supplementary file 1 [file SupplementaryFile1.zip › Supplementary Material/Supplementary Table S2.DOCX]

Supplementary Material

**Supplementary Table 2: Model fit, structural path estimates, and explained variance for the exploratory observed-variable path analysis.**

**Panel A. Model information and fit**

| **Model characteristic** | **Value** |
| --- | --- |
| Model type | Exploratory observed-variable path analysis |
| Experimental unit | Culture flask |
| Sample size | 75 |
| Estimator | Robust maximum likelihood (MLR) |
| Population adjustment | Four contrast-coded population covariates in each equation |
| Robust chi-square | 9.266 |
| Degrees of freedom | 1 |
| chi-square p-value | 0.002 |
| CFI | 0.983 |
| TLI | 0.091 |
| RMSEA | 0.081 |
| SRMR | 0.014 |
| AIC | 744.803 |
| BIC | 846.773 |

**Panel B. Primary structural paths**

| **Predictor** | **Response** | **B** | **Robust SE** | **Standardized beta** | **p-value** |
| --- | --- | --- | --- | --- | --- |
| NaCl | Osmoprotection | 0.512 | 0.052 | 0.512 | <0.001 |
| NaCl | Antioxidant response | -0.491 | 0.108 | -0.491 | <0.001 |
| Osmoprotection | Antioxidant response | -0.050 | 0.148 | -0.050 | 0.737 |
| NaCl | Damage | 0.316 | 0.130 | 0.316 | 0.015 |
| Osmoprotection | Damage | -0.008 | 0.104 | -0.008 | 0.936 |
| Antioxidant response | Damage | 0.002 | 0.108 | 0.002 | 0.982 |
| NaCl | Morphological adjustment | -0.035 | 0.069 | -0.035 | 0.611 |
| Osmoprotection | Morphological adjustment | 0.011 | 0.091 | 0.011 | 0.907 |
| Damage | Morphological adjustment | -0.267 | 0.070 | -0.267 | <0.001 |
| NaCl | Performance | 0.427 | 0.101 | 0.425 | <0.001 |
| Osmoprotection | Performance | -0.328 | 0.127 | -0.326 | 0.010 |
| Antioxidant response | Performance | -0.296 | 0.089 | -0.295 | <0.001 |
| Damage | Performance | -0.213 | 0.130 | -0.212 | 0.102 |
| Morphological adjustment | Performance | 0.181 | 0.115 | 0.180 | 0.115 |

**Panel C. Explained variance**

| **Response variable** | **R-squared** |
| --- | --- |
| Osmoprotection | 0.756 |
| Antioxidant response | 0.551 |
| Damage | 0.591 |
| Morphological adjustment | 0.725 |
| Performance | 0.624 |

**Notes.** B is the unstandardized path coefficient; SE is the robust standard error; beta is the completely standardized coefficient. Osmoprotection was represented by standardized proline; antioxidant response by an equal-weighted composite calculated from z-standardized APX, CAT, and GuPX activities; damage by standardized MDA; morphological adjustment by the standardized root-to-shoot ratio; and performance by standardized dry biomass. NaCl was included as a standardized continuous predictor. All measurements were obtained at the end of the experiment; consequently, the paths represent conditional associations and should not be interpreted as causal or temporal effects.
